# Disentangling the unique associations of age, pubertal stage, and pubertal hormones with white matter microstructure in childhood and adolescence

**DOI:** 10.1162/IMAG.a.1270

**Published:** 2026-06-15

**Authors:** Mark Curtis, Anastasia Yendiki, Theresa Cheng, Adam Omary, John C. Flournoy, Sridhar Kandala, Ashley F.P. Sanders, Michael P. Harms, Leah H. Somerville, Deanna M. Barch

**Affiliations:** Department of Psychological and Brain Sciences, Washington University in St. Louis, MO, United States; Athinoula A. Martinos Center for Biomedical Imaging, Massachusetts General Hospital and Harvard Medical School, Boston, MA, United States; Department of Psychology and Center for Brain Science, Harvard University, Cambridge, MA, United States; Department of Psychiatry, Washington University School of Medicine, St. Louis, MO, United States

**Keywords:** puberty, hormones, white matter, development

## Abstract

Puberty is associated with hormone level changes that influence white matter development. It remains unclear how pubertal stage and pubertal hormones *uniquely* relate to white matter microstructure development. Further, it is unclear if white matter tracts develop along a hierarchical sensorimotor-association (S-A) axis, similar to other aspects of brain development. We used the Human Connectome Project in Development cross-sectional sample of 1,105 youth (aged 5–21 years) to investigate unique associations of sex, age, pubertal stage, DHEA, testosterone, estradiol, and progesterone with fractional anisotropy (FA) within canonical white matter tracts. The average S-A axis rank was calculated for tract cortical endpoints. Age was the best-fit model for 17 tracts with prefrontal, parietal, and temporal connections. The Pubertal Timing model (age-adjusted pubertal stage) was the best fit for the superior longitudinal fasciculus 1. The DHEA model was the best fit for the splenium, genu, and prefrontal body of the corpus callosum. The Estradiol model was the best fit for the ventral cingulum bundle, extreme capsule, inferior longitudinal fasciculus, and uncinate fasciculus. The Full model was the best fit for the rostrum. Radial diffusivity explained more FA variability than axial diffusivity in all tracts. Pubertal Timing and DHEA were more related to FA in association-related tracts than sensorimotor-related tracts. Thus, during childhood and adolescence, Pubertal timing and DHEA may be uniquely related to white matter tract microstructure in association-related tracts.

## 1 Introduction

Adolescence, roughly spanning the ages of 10–24, is a developmental period associated with many biological, social-emotional, and societal role changes (Pfeifer & Allen, 2021; Sawyer et al., 2018). Puberty is the developmental process that is a catalyst for the transition from childhood to adolescence and is characterized by distinct but interacting changes in adrenal (adrenarche) and gonadal (gonadarche) hormone levels. Adrenarche is the early phase of puberty that begins in childhood and continues alongside gonadarche. It is associated with increases in dehydroepiandrosterone (DHEA) and changes in skin and hair. Gonadarche is associated with increases in testosterone, estradiol, and progesterone and changes in secondary sex characteristics. Pubertal processes influence numerous ongoing changes in brain structure and functioning that individuals experience during adolescence (Curtis et al., 2024). The brain’s white matter is one aspect of neurodevelopment that continues to develop during adolescence. However, the unique impact of pubertal processes on the development of white matter, versus chronological age, remains relatively understudied.

In general, white matter develops rapidly in infancy and early childhood with more subtle developmental changes throughout adolescence and young adulthood. White matter volume and myelination increase rapidly early in infancy but do not peak until the late 30s (Lebel & Deoni, 2018; Ouyang et al., 2019; Sowell et al., 2004). Measures extracted from diffusion-weighted imaging (DWI), such as fractional anisotropy (FA), radial diffusivity (RD), and axial diffusivity (AD), are known to be associated with microstructural features of white matter (myelination, fiber density, etc.) (Song et al., 2002, 2003, 2005; Takahashi et al., 2000). FA measures overall anisotropy, or directional coherence of water diffusion. As axons thicken from myelination of directionally coherent fibers, diffusion becomes highly restricted in the direction perpendicular to the fiber orientation, or highly anisotropic, leading to an increase in FA (Alexander et al., 2007). RD measures water diffusion perpendicular to the direction of the axons and is related to axonal packing, myelination, and diameter, while AD measures diffusion parallel to axons and is related to axonal integrity (Genc et al., 2023; Paus & Toro, 2009; Pesaresi et al., 2015; Song et al., 2002, 2005). These microstructural components follow a similar trajectory to white matter volume, as myelin and axonal density generally increase with age until the late 30s (Sowell et al., 2003; Toga et al., 2006). This is captured by DWI measures, with FA values generally peaking in the mid-30s, while RD and AD decrease during adolescence and young adulthood. However, the trajectories and peaks differ between different white matter tracts in the brain, as frontal-temporal connected tracts have the most protracted development (Lebel & Deoni, 2018). Although changes are more subtle than early life, white matter microstructure continues to be refined throughout adolescence and young adulthood with the influence of genetics and other processes, such as puberty.

Although pubertal mechanisms are likely related to the development of white matter, the nature of these relationships in humans is unclear. In humans, pubertal changes can be easily measured by changes in physical characteristics (e.g., skin, hair, height). Perceived physical changes can be reported by individuals or their parents to provide a subjective index of pubertal development. While not a direct measurement of physiological pubertal changes, these physical changes are related to the underlying changes in hormone levels (Shirtcliff et al., 2009). To measure puberty-specific effects relative to one’s age, pubertal stage can be statistically controlled for age. This is functionally equivalent to measuring pubertal timing, defined here as the progression of an individual’s puberty relative to individuals of a similar age (Bramen et al., 2012; Koolschijn et al., 2014; Peper et al., 2009). While a relationship between pubertal timing and FA is not always found (Chahal et al., 2018; Pohl et al., 2016), early pubertal timing appears to be related to less FA in the corticospinal tract, corpus callosum, and frontal cortex (Ando et al., 2021; Herting et al., 2012). Radial diffusivity is inversely related to pubertal stage (not age-corrected) in the corpus callosum, anterior thalamic radiation, superior longitudinal fasciculus, and uncinate fasciculus (Asato et al., 2010). Radial diffusivity, but not axial diffusivity, may be inversely related to pubertal timing in the insula (Herting et al., 2012). Thus, some evidence suggests that physical pubertal stage is related to white matter microstructure, but results are often mixed, and it remains inconclusive which tracts are particularly impacted by puberty. Further, it is unclear which aspects of puberty (e.g., physical pubertal status versus hormones) are related to white matter development.

While changes in pubertal hormones are linked to the physical changes in pubertal maturation, there remain differences between objectively measured hormone levels and the subjective physical measures of puberty due to the cyclical nature of hormone levels and individual differences in the hormone levels associated with the physical changes that accompany puberty. However, hormonal measurements provide an objective index of pubertal processes not available from the subjective physical measures alone. Changes in pubertal hormones are one aspect of puberty that seems to be related to white matter microstructural development. In rodent models, increases in pubertal hormones such as testosterone and estradiol are related to increases in oligodendrocyte number and myelin protein levels in frontal cortex and corpus callosum (Cerghet et al., 2006; Darling & Daniel, 2019). However, the direction of this relationship can depend on the location in the brain, as increases in estradiol are related to decreases in myelinated axons in the splenium (Yates & Juraska, 2008). In humans, the relationship between hormones and white matter microstructure is less clear. Age-adjusted testosterone levels appear to be positively related to FA in the superior temporal gyrus and frontal areas, and both FA and AD in the corpus callosum (Herting et al., 2012; Ho et al., 2020). Age-adjusted testosterone is inversely related to FA in the cerebellar peduncle and other subcortical-frontal connections (Herting et al., 2012; Peper et al., 2015). Age-adjusted DHEA levels are positively associated with RD in the corpus callosum (Barendse et al., 2018). Age-adjusted estradiol levels appear to be negatively related to FA in parietal regions and inferior fronto-occipital fasciculus (Herting et al., 2012; Stoica et al., 2019). While some of these studies suggest relationships between hormones and white matter microstructure, the findings are quite mixed. Many of these previous studies were in relatively narrow age ranges (total age range: 8–18), in relatively smaller samples (n’s <100), and there is inconsistent methodology on whether microstructural measures were measured from entire white matter tracts or parts of tracts (Barendse et al., 2018; Herting et al., 2012; Ho et al., 2020; Stoica et al., 2019). Thus, a more comprehensive understanding of how puberty, particularly beyond age, relates to white matter microstructure is needed. Examining multiple hormones in the same individuals, with the same methodology for assessing white matter microstructure allows for a more direct comparison.

As noted above, FA, the most commonly reported DWI microstructural measure is a general measure of anisotropy. Thus, many different mechanisms can cause changes in FA values. For example, an increase in FA could result from microstructural changes that impact RD or AD or a combination of the two. Some evidence suggests developmental changes in FA are mainly attributed to changes in RD (Faria et al., 2010; Giorgio et al., 2008; Lebel & Beaulieu, 2011), which may reflect increased myelination or enlargement of fibers. However, some studies suggest that both RD and AD both contribute to the FA changes (Ashtari et al., 2007). As both of these continue to change throughout development, but capture different aspects of underlying white matter, it is unclear to what degree these relate to the FA changes often reported in development. In addition, much of the previous work was in smaller samples, making it difficult to robustly assess the empirical relationship of RD and AD with FA. Here, we examined how much unique variance RD and AD contributed to changes in FA within various white matter tracts to better understand these relationships.

Finally, while many developmental changes in gray matter, functional connectivity and plasticity appear to progress along a hierarchical sensorimotor-association (S-A) axis (Sydnor et al., 2021), it is unknown how white matter development relates to the S-A axis. This axis spans from primary sensory and motor cortex to transmodal association cortex and is thought to be caused by a hierarchical critical period progression in which sensorimotor areas undergo critical period maturation early to deliver reliable outputs to cortical areas at the next level of cortical hierarchy (Larsen et al., 2023). Pubertal mechanisms may be related to this organization, as pubertal stage and pubertal hormones appear to substantially influence association cortex development (Curtis et al., 2024; Sydnor et al., 2021). Some evidence suggests that association tracts reach peak maturation later than commissural and projection pathways (Lebel & Deoni, 2018). In addition, white matter microstructure may mature sooner in sensory visual areas and later in more frontal areas, supporting a pattern of white matter development (Asato et al., 2010; Colby et al., 2011). Developmental myelination changes also differ systematically along the S-A axis, with sensory and motor cortices experiencing greater and more rapid myelination earlier (Baum et al., 2022). However, it is unknown if the relationships between pubertal timing or hormones and white matter microstructure in tracts follow an S-A axis pattern similar to other aspects of brain development.

Some evidence previously described suggests that pubertal stage and pubertal hormones are related to white matter microstructure. However, many of these studies either focus on different age ranges throughout adolescence (example age ranges: 10–16 years, 13–21 years, and 7–18 years), or include relatively smaller sample sizes (n’s < 100) (Ashtari et al., 2007; Barendse et al., 2018; Faria et al., 2010; Giorgio et al., 2008; Herting et al., 2012; Ho et al., 2020; Stoica et al., 2019). This leaves gaps in the understanding of patterns across the full course of school age and adolescent development. This is particularly important as childhood and adolescence is an especially vulnerable time to develop psychopathology, which is thought to be, in part, related to disruptions in white matter development. Thus, a better understanding of this critical literature gap on the role of puberty and pubertal hormones in typical microstructure development is necessary to understanding potential relationships to psychopathology (Kessler et al., 2005).

To address these gaps in the literature, we used the Human Connectome Project in Development large cross-sectional cohort from 1,105 youth (ages 5–21) to examine relationships between FA and age in canonical white matter tracts. Further, we examined whether pubertal hormones explained additional variance in white matter microstructure than more easily and commonly obtained variables of age and pubertal status. In addition, we examined how much unique variance RD and AD explained in FA development. Finally, we examined if the relationships between FA and the pubertal variables reflect variation along the sensorimotor-association axis.

## 2 Materials and Methods

### 2.1 Participants

Many of the participants and methods utilized here are similar to those reported in Curtis et al. (2024). This study utilized cross-sectional (visit 1) data from 1,264 youth aged 5–21 years from the Human Connectome Project in Development (HCP-D) (Harms et al., 2018; Somerville et al., 2018). Participants were recruited from four sites: Harvard University, University of California-Los Angeles, the University of Minnesota, and Washington University in St. Louis. The study was approved by the Institutional Review Board at Washington University in St. Louis. Participants provided written informed consent or assent, and parents of participants under 18 years old provided written informed consent for the child’s participation. Detailed inclusion criteria are provided in the Supplementary Data. Only participants with complete puberty, hormone, and diffusion imaging data were included in this analysis, and individuals with high motion were excluded, leaving a final dataset of 1,105 participants (see Supplementary Data for missing data specifics). Demographic characteristics for this final sample are provided in Table 1.

**Table 1.** Demographic and acquisition site characteristics of the sample.

	Participants (N = 1,105)
Mean age in years (SD)	13.3 (4.54)
Age range in years	5.01–21.9
Sex (% female)	50.8
Race (%)	
Native American/Alaska Native	0.2
Asian	6.8
Black/African American	12.1
Hawaiian or Pacific Islander	0.2
White	61.3
More than one race	17.0
Unknown	2.4
Median income ($)	110,000
Acquisition site (%)	
Harvard	27.3
UCLA	25.1
UMinn	26.2
WUSTL	21.4

Abbreviations: SD, standard deviation; UCLA, University of California Los Angeles; UMinn, University of Minnesota; WUSTL, Washington University in St. Louis.

### 2.2 Pubertal stage scales

The Morris-Udry scale (Morris & Udry, 1980) and the Pubertal Development Scale (PDS) (Petersen et al., 1988) were both collected for each participant to measure self-reported markers of physical development and secondary sex characteristics. The PDS scores were adjusted to a 5-point scale to parallel the five Tanner stages, the standard system for describing the physical changes associated with pubertal development (Shirtcliff et al., 2009). The scores from the individual questions on the Morris-Udry and PDS were averaged and converted to z-scores. The two z-scores were averaged to calculate a singular pubertal composite score (additional details in Curtis et al., 2024; Omary et al., 2025).

### 2.3 Hormone acquisition and processing

Participants filled a Salimetrics SalivaBio passive drool kit at home in the morning, before eating, drinking, and brushing their teeth. Males, premenarcheal females, and menarcheal females without regular cycles were instructed to collect saliva samples on the morning of their first session. Postmenarcheal females with regular cycles were instructed to collect the sample during the early follicular phase (cycle day 7) when hormone levels are relatively stable and low (Koolschijn et al., 2014). While time since waking for the saliva collection was not collected in this dataset, the HCPD protocol asked participants to collect samples within 30 minutes of waking. Collection time was reported, and mean collection time in this sample was 8:16 am ± 108 minutes. Samples were frozen at home, transported to the study site in a freezer pack on the day of appointment, and frozen at the study site (< -70°C). Saliva samples were processed with a standard ELISA in a single batch. Salimetrics immunoassay was used to assay DHEA, testosterone, estradiol, and progesterone in duplicate. The final values are the average of the two replicates (Herting et al., 2020). The mean inter-assay CVs between plates were 14.77% for testosterone, 14.33% for DHEA, 11.08% for estradiol, and 13.42% for progesterone. The mean intra-assay coefficient of variations (CVs) between replicates was 6.01% for testosterone, 5.98% for DHEA, 5.19% for estradiol, and 5.03% for progesterone. If the assay produced an undetectable value below the lower sensitivity threshold, the hormone value was set to 0 (DHEA: N = 28, Testosterone: N = 29, Estradiol: N = 67, Progesterone: N = 8), in agreement with ABCD Study protocols (Herting et al., 2020). Detectable measurements falling below the lower sensitivity threshold were kept and deemed interpretable, per expert guidance. Since normally distributed data is not a requirement for Generalized Additive Models (GAMs), raw final hormone values were used. Hormonal medications, other than contraceptives, were an exclusion criterion for the study. Since hormonal birth control impacts hormone levels, hormonal contraceptive use was included as a covariate for the main analyses. In addition, sensitivity analysis was conducted that excluded those individuals to examine their potential impact on the results. Additional details on saliva hormone data and detailed analyses of pubertal measures and hormones in relation to age, sex, and their interaction from this dataset are reported in Omary and colleagues and Curtis and colleagues (Curtis et al., 2024; Omary et al., 2025).

### 2.4 MRI acquisition

Structural MRI data were acquired on a Siemens 3T Prisma scanner with a 32-channel head coil. Sagittal T1-weighted MR images were obtained with a multi-echo 3D MPRAGE sequence [TR/TI = 2500/1000 ms, TE = 1.8/3.6/5.4/7.2 ms, flip angle = 8^o^, FOV = 256 x 240 x 166 mm, matrix size = 320 x 300, 0.8 mm isotropic voxels, 208 slices, GRAPPA acceleration factor = 2]. T2w volumes were acquired at the same spatial resolution using a variable-flip-angle turbo-spin-echo 3D SPACE sequence (Mugler et al., 2000) [TR/TE = 3200/564 ms; FOV = 256 x 240 x 166 mm, matrix = 320 x 300, 0.8 mm isotropic voxels]. Diffusion-weighted data were collected using an echo-planar diffusion-weighted sequence (repetition time = 3,230 ms, echo time = 89.2 ms, field of view = 210 mm, voxel size = 1.5 × 1.5 × 1.5 mm^3^, flip angle = 78, multi-band factor = 4, with no in-plane acceleration). The diffusion MRI data were acquired as four ~5.5 minutes dMRI runs with 185 diffusion-weighting directions sampled on the whole sphere, each acquired twice with opposite phase encoding direction (AP/PA) to facilitate robust correction of distortions. These images are acquired with a 2-shell protocol at b-values of 1500 and 3000 s/mm^2^. The shells were interleaved within each dMRI run, and 28 b = 0 s/mm^2^ volumes were equally interspersed across the four runs (Harms et al., 2018).

### 2.5 MRI processing

The structural T1w and T2w scans were processed using HCP pipelines as described previously (Curtis et al., 2024) to create the prerequisite anatomical information for FreeSurfer’s TRACULA (Maffei et al., 2021; Yendiki et al., 2011, 2014). The diffusion data were first preprocessed using the “DiffusionPreprocessing” stream of HCP Pipelines (v4.0.1) (Glasser et al., 2013, 2016). This pipeline includes intensity normalization, susceptibility distortion correction (via FSL’s ‘topup’) (Andersson et al., 2003), and correction for eddy current distortions and motion via FSL’s ‘eddy’ tool (parameters in Supplementary Data) (Andersson & Sotiropoulos, 2016). We used the advanced ‘eddy’ features of outlier replacement (Andersson et al., 2016), slice-to-volume motion correction (Andersson et al., 2017), and correction for susceptibility-by-movement interactions (Andersson et al., 2018). The b-vectors were rotated to account for motion (Sotiropoulos et al., 2013). Finally, the dMRI data was corrected for gradient nonlinearity distortion as part of resampling to the subject’s native T1w space from the HCP structural pipeline output (while maintaining the same 1.5 mm spatial resolution of the dMRI data).

Data were then processed with FSL’s ‘bedpostx’ (Behrens et al., 2007; Hernandez-Fernandez et al., 2019) to estimate the diffusion distribution at each voxel. Bedpostx was run modeling up to 3 fibers per voxel (Sotiropoulos et al., 2016; Yendiki et al., 2011), using the deconvolution model with zeppelins (‘model = 3’), assuming rician noise, and set to account for the effect of the gradient nonlinearities on the strength and direction of the diffusion-sensitizing gradients at each voxel (Sotiropoulos et al., 2013). The data for individuals with absolute and relative motion outliers determined by the “SQUAD” report (Bastiani et al., 2019) were visually inspected to determine the data showed reduced distortion and motion artifacts after it was corrected for susceptibility, eddy currents, and subject movement using a combination of topup and eddy.

TRACULA (TRActs Constrained by UnderLying Anatomy) is a tool that automatically reconstructs a set of major white-matter pathways from diffusion-weighted MRI data by fitting the pathway both to the fiber orientation vectors derived from the individual’s diffusion MRI data, and to the anatomical neighborhood priors derived from the individual’s T1w MRI data (Maffei et al., 2021; Yendiki et al., 2011). TRACULA was used to generate the probability distributions for 42 canonical white matter pathways by simultaneously fitting the shape of each pathway to the results from the ball-and-stick model (from bedpostx) and to the prior information of the pathway from the manually annotated TRACULA atlas. Microstructural maps were computed using all shells. Average values for FA, RD, and AD were computed for each individual for each tract of interest by averaging values across the voxels that remained after thresholding the posterior probability distribution of that tract at 20% of its maximum. Tracts were visually inspected for partial or failed tract reconstructions, and TRACULA was rerun on tracts with partial or failed reconstructions (Maffei et al., 2023). TRACULA was rerun by reinitializing the control points of the initial spline (‘reinit = 1’) (Yendiki et al., 2011). Individuals with tracts that had partial or failed reconstructions after reinitialization were excluded from subsequent analyses (n = 4 participants). Measures from the left and right hemispheres for the 16 bilateral tracts were averaged.

### 2.6 Total motion index

For each participant, a total motion index (TMI) score was calculated as previously described (Yendiki et al., 2014). TMI is a composite score of four motion measures calculated in TRACULA: 1) average volume-by-volume translation (degrees), 2) average volume-by-volume rotation (mm), 3) percent of slices with excessive intensity drop-out, and 4) average drop-out score for slices with excessive intensity drop-out. To obtain these values, “trac-all -qa” was run on the original DWI data (i.e., prior to preprocessing through ‘eddy). For each subject, the TMI was computed with the following formula:

$$TMI≜\sum_{j=1}^{4}\;\frac{x_{ij}\;-M_{j}}{Q_{j}\;-q_{j}}$$

where 𝑗 = 1,⋯,4 are the four motion measures listed above, 𝑥_ij_ is the value of the *j*-th motion measure for the *i*-th subject and 𝑀_𝑗_, 𝑄_𝑗_, and 𝑞_𝑗_ are respectively the median, upper quartile, and lower quartile of the *j*-th measure over all the subjects. TMI was negatively correlated with age (r = -0.34). Individuals with high motion were excluded (± 3 standard deviations of the mean Total Motion Index, n = 26), and TMI was included as a covariate in the models.

### 2.7 Analysis plan

We began by first examining the associations of FA with age, and then as described in more detail below, examined the added relations to pubertal stage and hormones. To do so, we used generalized additive models (GAM) using the “gam” function within the “mgcv” package (version 1.8-38) (Wood, 2001) in R (version 4.4.0) (R Development Core Team, 2022). Using penalized splines, GAMs can flexibly capture linear and nonlinear relationships. Nonlinearities were estimated using restricted maximum likelihood (REML), where smooth terms are penalized for increasing complexity. The age associations with FA were tested for nonlinearity by including a second-order penalty (specified with (m = c(0,2)) within the Age smooth) with the linear Age term included in the model as well. If the smooth term is significant, it suggests a non-linear fit which could not be modeled by the corresponding linear model. Significance was determined if the Age smooth was *p* < 0.05. The following model was used to determine significant nonlinear fits for each tract.

**Model:** FA ~ Age + s(Age, m = c(2,0)) + Site + Total Motion Index

The following model was used to plot the age associations with FA in each tract.

**Model:** FA ~ s(Age) + Site + Total Motion Index

Derivatives of the Age smooth for each tract determined to have a significant nonlinear fit were calculated with the “derivatives” function in the “gratia” package. Periods of significant change were calculated and plotted with simultaneous confidence intervals, which are calculated from simulations of the maximum absolute standardized deviations of the fitted model from the true model (1000 simulations, 95%). Significant change was determined as where the simultaneous interval on the derivative excluded 0.

### 2.8 Unique contributions of age, pubertal stage, and hormones to FA

GAMs were again used to model FA for each white matter tract as predicted by sex, age, pubertal stage, and hormone levels. Default “mgcv” smoothing parameters were used, such as thin plate regression splines, and the basis dimension (k) was left at the default value of 10, which indicates the maximum effective degrees of freedom (EDF) the model smooth could use. This analysis was used to investigate the additional contribution of pubertal stage above sex and age and the additional contribution of pubertal hormones above sex, age, and pubertal stage. The effects of sex were modeled as the “baseline” model, followed by a model that included both sex and age. Beyond including a main effect of sex, we included factor-smooth interactions between age and sex to allow for more flexible models in which each sex could have separate age-dependent trajectories.

Pubertal stage scores were then added to the sex and age model. In this study, we operationalized pubertal timing as pubertal stage adjusted for age and sex. Thus, this “Pubertal Timing Model” includes pubertal stage, age, and sex. This model tests the relationship between FA and the pubertal stage relative to same aged and sex peers (i.e., pubertal timing). When both age and pubertal stage are included in the same model, the model fit is functionally equivalent to a commonly used method to obtain pubertal timing, which is defining a pubertal timing term by extracting the residuals from a model that regresses puberty on age by sex (Dorn et al., 2006). In this study, we are focused on whether a pubertal timing model (with pubertal stage, age and sex all included as dependent variables) explains more variance than a model with just age and sex. Each hormone was then added individually to the sex, age, and pubertal timing model to investigate the additional contribution of each hormone above and beyond sex, age, and pubertal stage. Finally, a full model included all variables. Each model included acquisition site, birth control use (yes/no), and the total motion index as a covariate. The following models were used:

**Sex:** FA ~ Sex + Site + Birth Control + Total Motion Index
**Age:** FA ~ Sex + s(Age) + s(Age, by = Sex) + Site + Birth Control + Total Motion Index
**Pubertal Timing:** FA ~ Sex + s(Age) + s(Age, by = Sex) + s(Pubertal Composite) + s(Pubertal Composite, by = Sex) + Site + Birth Control + Total Motion Index
**DHEA (DH):** FA ~ Sex + s(Age) + s(Age, by = Sex) + s(Pubertal Composite) + s(Pubertal Composite, by = Sex) + s(DH) + s(DH, by = Sex) + Site + Birth Control + Total Motion Index
**Testosterone (TE):** FA ~ Sex + s(Age) + s(Age, by = Sex) + s(Pubertal Composite) + s(Pubertal Composite, by = Sex) + s(TE) + s(TE, by = Sex) + Site + Birth Control + Total Motion Index
**Estradiol (ES):** FA ~ Sex + s(Age) + s(Age, by = Sex) + s(Pubertal Composite) + s(Pubertal Composite, by = Sex) + s(ES) + s(ES, by = Sex) + Site + Birth Control + Total Motion Index
**Progesterone (PR):** FA ~ Sex + s(Age) + s(Age, by = Sex) + s(Pubertal Composite) + s(Pubertal Composite, by = Sex) + s(PR) + s(PR, by = Sex) + Site + Birth Control + Total Motion Index
**Full Model:** FA ~ Sex + s(Age) + s(Age, by = Sex) + s(Pubertal Composite) + s(Pubertal Composite, by = Sex) + s(DH) + s(DH, by = Sex) + s(TE) + s(TE, by = Sex) + s(ES) + s(ES, by = Sex) + s(PR) + s(PR, by = Sex) + Site + Birth Control + Total Motion Index

A model comparison approach was utilized to first determine if the sequential addition of age, pubertal stage, and each hormone resulted in a better fitting model relative to the simpler model lacking the added term. A better fitting model was determined by an Akaike information criterion (AIC) value two units less than the simpler model (Bozdogan, 1987). Using a threshold of 2 AIC units provides more confidence in the winning model among the multiple comparisons (Burnham & Anderson, 2002). The best fit model was determined as the most complex model with an AIC 2 units less than the simpler models. This comparison continued through all models, even if the 2 AIC unit criteria was not satisfied for an earlier comparison in the progression. For example, if for a given network, the pubertal timing model did not have an AIC 2 units less than the age model, but the DHEA model had an AIC 2 units less than the age and pubertal timing model, the DHEA model would be determined as the best fitting model.

Then, to examine the additional contribution of pubertal stage above sex and age and the additional contribution of pubertal hormones above sex, age, and pubertal stage, the adjusted R^2^ change was calculated sequentially between the more complex model and the simpler model (e.g., Age-Sex, Pubertal Timing-Age, {DH,TE,ES, or PR2-Pubertal Timing, and Full-Pubertal Timing).

Finally, since these puberty-related variables are correlated (Spearman’s Rho values = 0.9–0.14, Supplemental Table S1 & Supplemental Fig. S1), we wanted to determine the unique variance that each variable included in this study (e.g., sex, age, pubertal stage, hormone) contributed to FA in each tract. To do this, a partial R^2^ was calculated. This was calculated for each variable in the full model by taking the difference between R^2^ of the full model and a reduced model lacking that specific variable. For example, to determine the partial R^2^ for age, the difference was calculated between the R^2^ of the full model and the R^2^ of a reduced model without age (i.e., full model with age removed). This provided an effect size for how much unique variance was accounted for by each term (age, pubertal stage, or pubertal hormone) when simultaneously controlling for all the other factors (reported as a percentage). The pubertal stage term itself (which is dropped in the reduced model when calculating the partial R^2^ for pubertal stage) has no age or sex adjustment. However, the partial R^2^ for pubertal stage is a measure of unique variance after adjusting for age, sex, and all four hormones. This is in contrast to our discussion of the models themselves in the model comparison analysis (see above), where the pubertal timing model includes sex and age.

### 2.9 Interactions with sex

For each model that was the best fit to the data, we tested whether the interactions with sex for each of the terms in the winning model were significant. To do this, the “sex” term was classified as an ordered factor in the best fitting GAM model for that tract, which estimates a smooth for the first level of each term (female) and a difference smooth for the second level of each term (male). The difference smooth term was considered significant if *p* < 0.05. Benjamini-Hochberg False Discovery Rate (FDR) correction (*q* < 0.05) (Benjamini & Hochberg, 1995) was applied to correct for multiple comparisons for all interactions terms in all the best-fitting models.

### 2.10 Sensorimotor-association axis

We investigated the relationships between the variance added for each model of interest (Sex, Age, Pubertal Timing, DHEA, Testosterone, Estradiol, & Progesterone) and the sensorimotor-association (S-A) axis rank for each tract. Since the S-A axis has been characterized on the cortical surface, we used the cortical endpoints of the white matter tracts for this ranking. Cortical vertices ranked according to the sensorimotor-axis were obtained from GitHub (https://github.com/PennLINC/S-A_ArchetypalAxis) (Sydnor et al., 2021). The surface label files of the tract endpoints for all individuals were converted to label gifti files with Freesurfer’s “mris_convert”. Those files were resampled to the 32k_fs_LR standard surface using the workbench command “wb_command –label-resample”. Those files were then merged across all participants (“wb_command -label-merge”). Probability maps for the cortical endpoints for each tract were then calculated using the “wb_command -label-probability”. Note the middle cerebellar peduncle tract from TRACULA does not have cortical endpoints and thus was not included in this analysis. Vertices in which the probability was greater than 25% were retained as a group-derived probability mask for each endpoint (Storsve et al., 2016).

The goal was to relate the FA variance explained by each variable in each tract to the S-A axis. Thus, each tract was given one rank on the S-A axis based on the average S-A axis value within the tract’s two endpoint masks. The additional contribution of each term to the FA variance was used as previously described (i.e., adjusted R^2^ change was calculated sequentially between the more complex model and the simpler model). Then, for each model (Age, Pubertal Timing, etc.) correlations were used to test the relationship between the adjusted R^2^ change in FA and average S-A ranking value of each tract.

Finally, to directly compare the relationships between Age and Pubertal Timing with the S-A axis, the Age adjusted R^2^ change was calculated as the difference between the Pubertal Timing model and a reduced model without Age. Then, the Age and Pubertal Timing correlations were tested for a significant difference. The Steiger’s method in the “cocor” package in R with dependent correlation coefficients was utilized to determine if correlation coefficients of Age and Pubertal Timing were significantly different from each other (Diedenhofen & Musch, 2015). Correlations and correlation differences were considered significant at *p* < 0.05. This allowed for the comparison of the relationship between FA variance explained by each model and the average S-A rank of the tracts.

### 2.11 FA variance explained by RD and AD

The variance of FA explained by radial and axial diffusivity was determined by setting FA as the dependent variable and RD (or AD) as the independent variable, along with covariates in the model. The adjusted R^2^ value was extracted from these models.

**RD Model:** FA ~ RD + Site + Total Motion Index
**AD Model:** FA ~ AD + Site + Total Motion Index

### 2.12 Supplemental analyses

#### 2.12.1 Age-adjusted hormone models

While the main analysis was to determine if hormones explained meaningful unique additional variance in FA above and beyond both age and pubertal stage, it was of interest to also examine if any pubertal variable explained additional variance above and beyond age without giving pubertal stage priority over the hormone measures. To accomplish this, a supplemental analysis was performed that treated pubertal stage and hormones similarly by adding them individually to the Age model, instead of including pubertal stage in the hormone models. Results are briefly described below and reported in more detail in Supplementary Data.

#### 2.12.2 Puberty-adjusted age models

Pubertal variables (e.g., stage and hormones) are typically not given priority over age in sequential modeling, rendering pubertal measures secondary to age (as in the models above), despite evidence for the causal influence of hormones on brain structure. Therefore, we conducted a supplemental analysis in which pubertal variables were instead given priority in the sequential modeling, with age added last (as part of the full model) to investigate the relative contributions of pubertal variables without age taking precedence. Results are briefly described below and reported in more detail in Supplementary Data.

#### 2.12.3 Restricted age range

The age window of active pubertal development is typically ~9–18 years old. To evaluate whether the inclusion of ages outside this window influenced the sensitivity to detect pubertal effects, a sensitivity analysis was conducted using just the restricted age range of 9–18 years (N = 670). Results are briefly described below and reported in more detail in Supplementary Data.

#### 2.12.4 Birth control sensitivity analyses

Fifty-nine individuals reported hormonal birth control use. Since hormonal birth control impacts hormone levels, a sensitivity analysis was conducted that excluded those individuals to examine their potential impact on the results. Results are briefly described below and reported in more detail in Supplementary Data.

## 3 Results

### 3.1 FA relationships with age

**Fig. 1.**
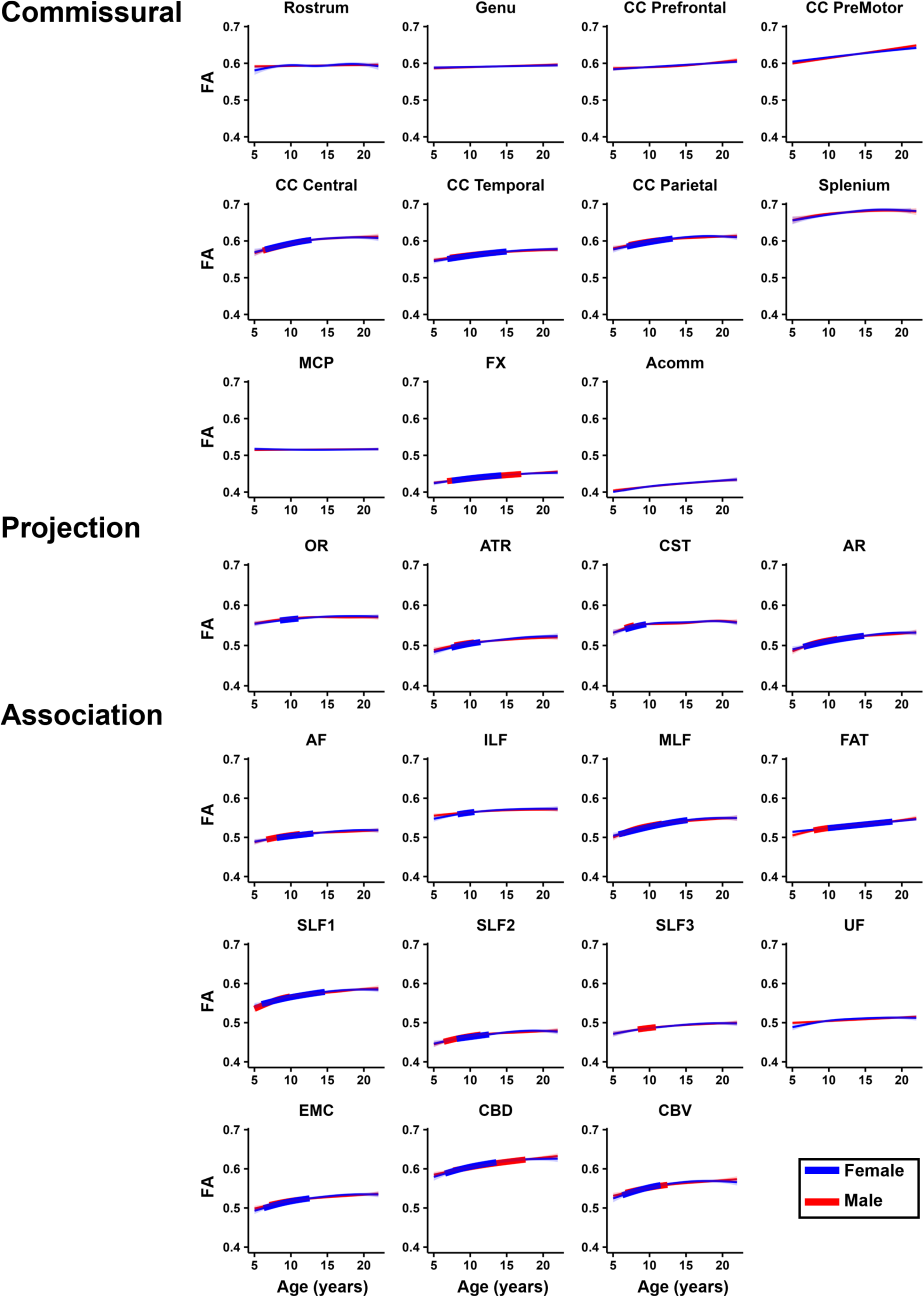
Associations between FA and Age. The relationships between age and FA in each canonical tract from TRACULA. Tracts are grouped into three main groups according to their projections. Commissural tracts are interhemispheric, Projection tracts project between cortex and subcortical regions or spinal cord, and Association tracts connect regions within the same hemisphere. Twenty-one of the 26 tracts were best fit by nonlinear models. Significant changes of the slope of the relationship between age and FA were calculated within each tract by simultaneous confidence intervals of the derivative and are highlighted. The anterior commissure, premotor body, genu, and rostrum of the corpus callosum had linear increasing trajectories. Tracts: Acomm, Anterior Commissure; CC, Corpus Callosum, which has the following sub-parts: Central Body; Parietal Body; Prefrontal body; Premotor body; Temporal Body; Genu; Rostrum; Splenium; AF, Arcuate Fasciculus; AR, Acoustic Radiation; ATR, Anterior Thalamic Radiation; CBD, Dorsal Cingulum Bundle; CBV, Ventral Cingulum Bundle; CST, Corticospinal Tract; EMC, Extreme Capsule; FAT, Frontal Aslant Tract; FX, Fornix; ILF, Inferior Longitudinal Fasciculus; MLF, Middle Longitudinal Fasciculus; OR, Optic Radiation; SLF, Superior Longitudinal Fasciculus; UF, Uncinate Fasciculus; MCP, Middle Cerebellar Peduncle.

FA and age had a significant nonlinear relationship in most tracts (20/26 tracts) (Fig. 1). Nearly all tracts were positively associated with age. FA in the middle cerebellar peduncle was not significantly associated with age. In tracts that showed nonlinear relationships with age, the periods of more rapid increase in FA tended to occur in the younger age range (~5–12 years old) compared to the older ages (Supplemental Fig. S2). The uncinate fasciculus, middle cerebellar peduncle, prefrontal body, premotor body, genu, and rostrum of the corpus callosum had linear relationships. Complete GAM output statistics for these FA-Age relationships are reported in Supplemental Table S2, and summary statistics for FA in each tract are reported in Supplemental Table S3.

### 3.2 FA model comparison

#### 3.2.1 Sex

**Fig. 2.**
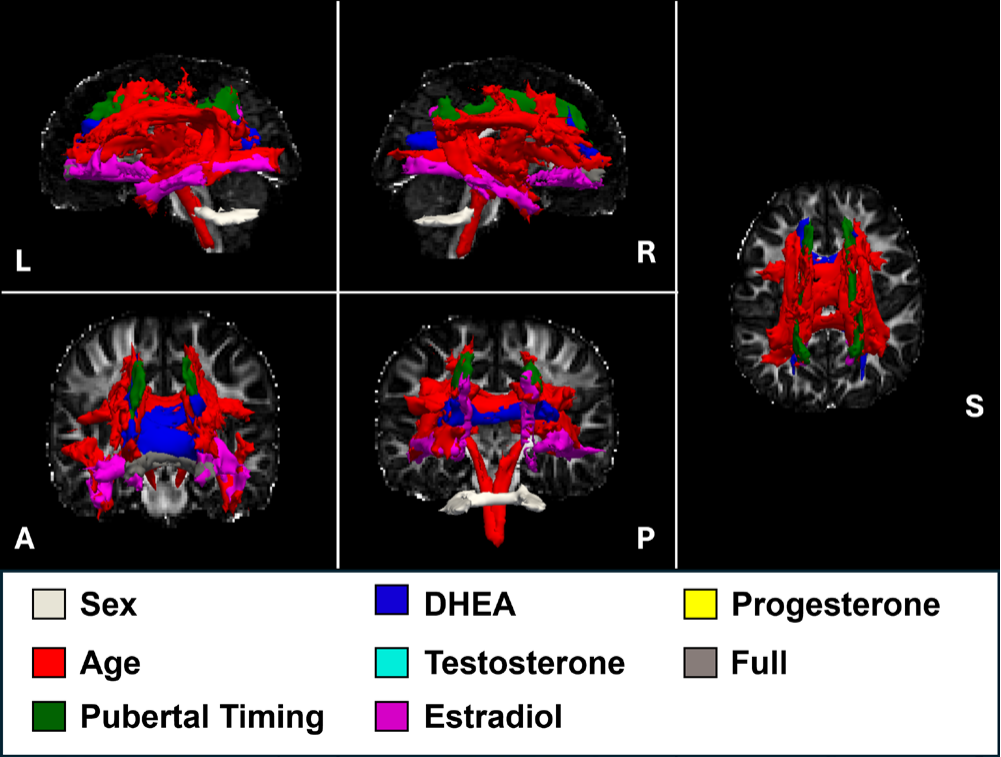
Best fit models for FA in each TRACULA tract. TRACULA was used to generate 42 canonical white matter tracts. For the 16 bilateral tracts, FA from the left and right hemispheres were averaged. The best fit model was determined as the most complex model with an AIC 2 units less than the simpler models. Note the models included additional terms sequentially. The Sex model included sex. The Age model included sex and age. The Puberty Timing model included sex, age, and pubertal stage. The four hormone models (DHEA, Testosterone, Estradiol, and Progesterone) included sex, age, puberty, and that specific hormone. The Full model included all terms. Sex was the best fitting model for the middle cerebellar peduncle. The Age model was the best fit model for 17 tracts. The Pubertal Timing model was the best fit for the superior longitudinal fasciculus I. The DHEA model was the best fit for the prefrontal body, splenium, and genu of the corpus callosum. The Estradiol model was the best fit for the ventral cingulum bundle, inferior longitudinal fasciculus, extreme capsule, and uncinate fasciculus. The Full model was the best fit for the rostrum of the corpus callosum. The Testosterone and Progesterone models were not the best fit for any tracts.

**Fig. 3.**
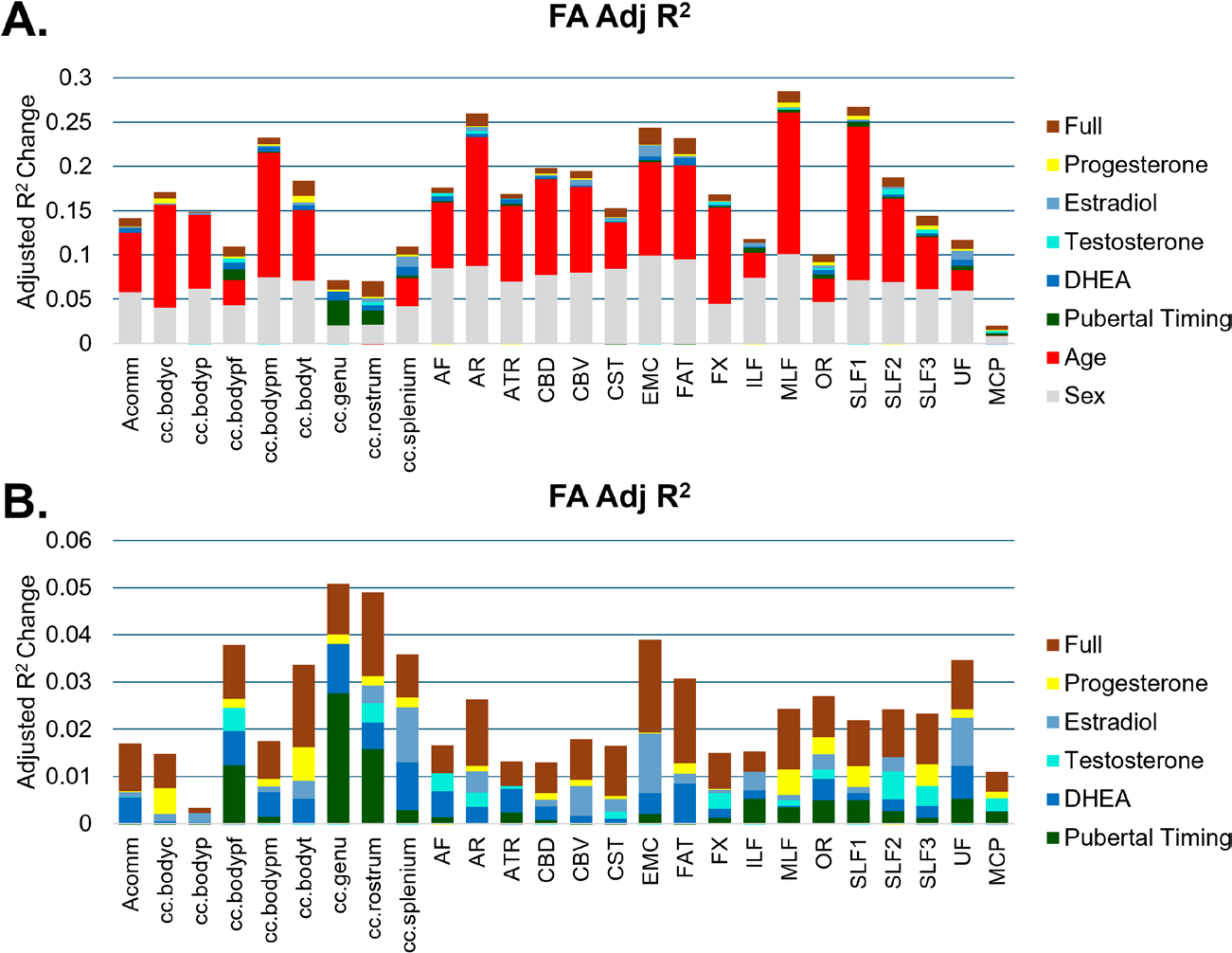
FA adjusted R^2^ Model Differences. Bars show the adjusted R^2^ for the baseline Sex model, and the *change* in adjusted R^2^ between the more complex and simpler model for the other variables (e.g., Age-Sex, Pubertal Timing-Age, {DHEA, Testosterone, Estradiol, or Progesterone}-Pubertal Timing, and Full-Pubertal Timing). Note the values for Hormone and Full models are the adjusted R^2^ change calculated from the Pubertal Timing model. They are not additive upon the other hormone models. For example, the yellow color is depicting the R^2^ change of the Progesterone model from the Pubertal Timing model, even though it is stacked on top of the DHEA, Testosterone, and Estradiol R^2^ change values. Consequently, the cumulative height of the bars should not be interpreted as an indication of the total variance explained by the Full model. (A) The addition of age accounted for the largest amount of additional variance. The pubertal timing and hormone models had smaller adjusted R^2^ changes in the models for most tracts. (B) The adjusted R^2^ change values for just the pubertal variables are plotted on a different scale to better visualize differences between tracts. Tracts: Acomm, Anterior Commissure; CC, Corpus Callosum, which has the following sub-parts: Bodyc, Central Body; Bodyp, Parietal Body; Bodypf, Prefrontal body; Bodypm, Premotor body; Bodyt, Temporal Body; Genu; Rostrum; Splenium; AF, Arcuate Fasciculus; AR, Acoustic Radiation; ATR, Anterior Thalamic Radiation; CBD, Dorsal Cingulum Bundle; CBV, Ventral Cingulum Bundle; CST, Corticospinal Tract; EMC, Extreme Capsule; FAT, Frontal Aslant Tract; FX, Fornix; ILF, Inferior Longitudinal Fasciculus; MLF, Middle Longitudinal Fasciculus; OR, Optic Radiation; SLF, Superior Longitudinal Fasciculus; UF, Uncinate Fasciculus; MCP, Middle Cerebellar Peduncle.

A sex only model was the best fitting model for only one tract, the middle cerebellar peduncle (Fig. 2), accounting for 1.3% unique variance (Fig. 3).

#### 3.2.2 Age

**Fig. 4.**
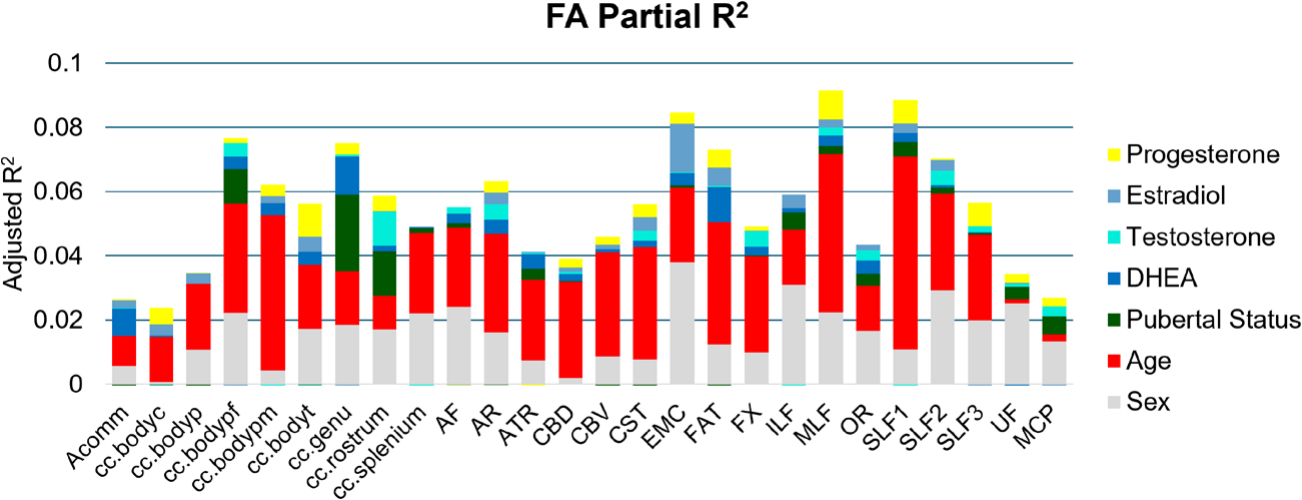
Unique variance explained in FA. A partial R^2^ was calculated for each variable in the full model by taking the difference between the full model and a reduced model lacking that specific variable. For example, to determine the partial R^2^ for age, the difference was calculated between the R^2^ of the full model and the R^2^ of a reduced model without age (i.e., full model with age removed). This provided an effect size for how much unique variance was accounted for by each term (age, pubertal stage, or pubertal hormone) when simultaneously controlling for all the other factors. Sex and Age explained the most unique variance in almost every tract. In the context of still less unique variance than sex and age, DHEA explained a relatively larger amount of unique variance in the genu and splenium. Similarly, estradiol explained a relatively larger amount of unique variance in the extreme capsule (EMC).

The model that included age in addition to sex explained a relatively large amount of additional FA variance in most tracts (except for the genu and rostrum) (Fig. 3). The Age model was the best fitting model for the following 17 tracts: central, parietal, premotor, and temporal aspects of the body of the corpus callosum; anterior commissure, fornix, anterior thalamic radiation, corticospinal tract, optic radiation arcuate fasciculus; acoustic radiation; dorsal cingulum bundle; frontal aslant tract; middle longitudinal fasciculus, and superior longitudinal fasciculi II, and III (Fig. 2), with age accounting for 0.9% to 6.0% unique variance in FA within these tracts (Fig. 4).

#### 3.2.3 Pubertal timing

The model that included pubertal stage in addition to sex and age is referred to as the pubertal timing model since it isolates effects of individual differences in pubertal timing relative to same-aged peers. This model explained a small amount of additional FA variance in most tracts (Fig. 3). To highlight differences between tracts, the added variance for the pubertal timing (and pubertal hormone) models is shown in Figure 3B. The Pubertal Timing model was the best-fitting model for the superior longitudinal fasciculus I (SLF1) (Fig. 2), with pubertal stage accounting for 0.5% unique variance (Fig. 4). Increased pubertal timing was related to more FA within the SLF1 at younger ages (~5–9 years old) and less FA at older ages.

#### 3.2.4 Pubertal hormones

Pubertal hormones explained a relatively small amount of additional variance of FA (Fig. 3). The DHEA model was the best-fitting model for the prefrontal body, splenium, and genu of the corpus callosum (Fig. 2), with DHEA accounting for 0.4%, 0.1%, and 1.2% unique variance respectively. Increased levels of DHEA, controlled for age and pubertal stage, are related to lower FA. The Estradiol model was the best-fitting model for the ventral cingulum bundle, inferior longitudinal fasciculus, uncinate fasciculus, and extreme capsule (Fig. 2), with estradiol accounting for 0.2%, 0.4%, 0.1%, and 1.5% unique variance respectively (Fig. 4). Increased levels of estradiol, controlled for age and pubertal stage, are related to lower FA. The Testosterone and Progesterone models were not the best fit for any tracts.

#### 3.2.5 Full model

The full model was the best fit for the rostrum of the corpus callosum (Fig. 2), with sex, age, and pubertal stage accounting for more unique variance than hormones (Fig. 4).

### 3.3 Contributions of RD and AD to FA

**Fig. 5.**
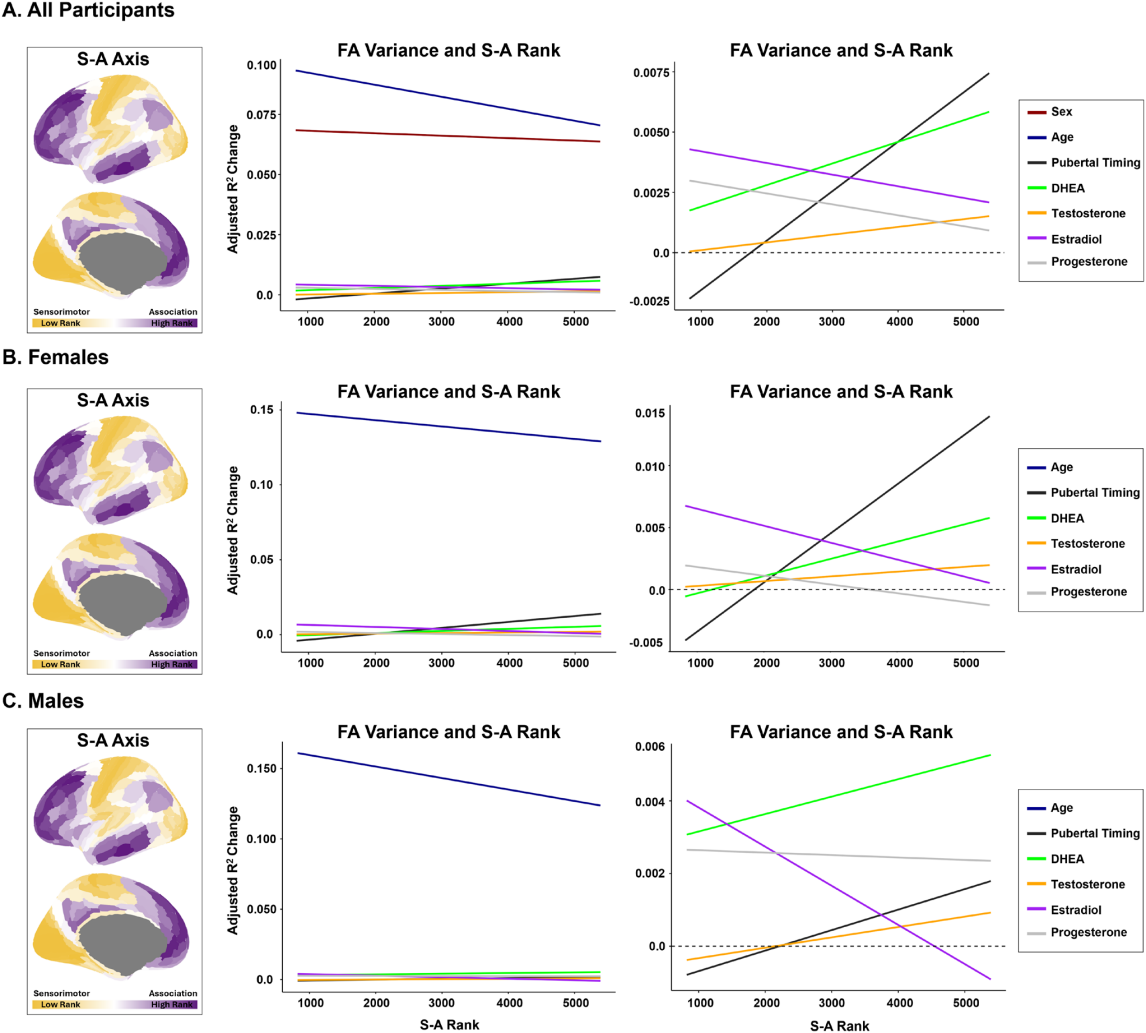
FA variance explained by pubertal terms and the sensorimotor-association axis. Many developmental changes appear to progress along a hierarchical sensorimotor-association axis, shown for reference on the left (Sydnor et al., 2021). Higher values on the S-A axis refer to higher association ranking, while lower values refer to higher sensorimotor ranking. (A) The fitted regression lines for the relationships between the adjusted R^2^ change for each model and the S-A value for each term are shown. Pubertal Timing (r = 0.43) and DHEA (r = 0.42) were significantly positively correlated with S-A value. Age (r = -0.14) showed a slightly negative correlation (nonsignificant) with S-A value. The right panels show the associations between the change in adjusted R^2^ and the S-A value for just the pubertal terms plotted on a different y-axis scale to better visualize these relationships. (B-C) The same relationships separated by females (B) and males (C).

To examine how much radial and axial diffusivity changes within white matter tracts contributed to the FA changes, the unique variance of RD and AD to FA values were measured. In all tracts, RD models explained more variance (58%-89%) than AD (5%-23%) (Fig. 5).

### 3.4 FA winning model endpoints and sensorimotor-association axis

**Fig. 6.**
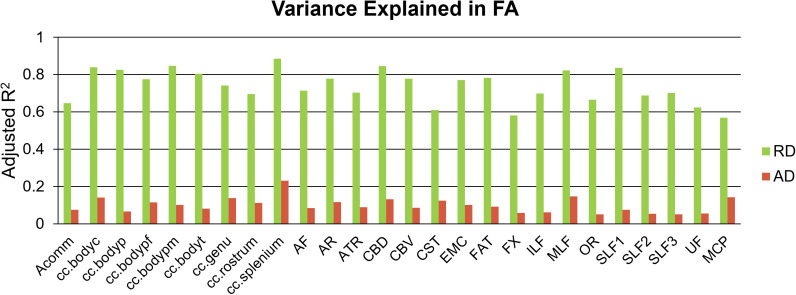
Radial and axial diffusivity contributions to FA. The unique FA variance explained by radial diffusivity (RD) and axial diffusivity (AD) was measured. In all tracts, RD models explained more FA variance than AD.

To examine whether the variance explained in each tract by age and pubertal terms differed on the S-A axis, the cortical endpoints of the tracts were assigned a ranking on the S-A axis (Supplemental Table S4). For each model (Age, Pubertal Timing, etc.), correlations were used to test the relationship between the adjusted R^2^ change in FA and the average S-A rank value of each tract. Pubertal Timing (r = 0.43) and DHEA (r = 0.42) were significantly positively correlated with S-A axis value (*p*’s < 0.05). No other terms were significantly correlated with S-A axis value, though it is of note that Sex (r = -0.06), Age (r = -0.14), Estradiol (r = -0.18), and Progesterone (r = -0.27) appeared to be slightly negatively correlated (*p*’s>0.05) (Fig. 6 & Supplemental Fig. S3).

### 3.5 Interactions with sex

For each model that was the best fit for each tract, it was tested whether the interactions with sex for each of the terms in the winning model were significant. There were no significant interactions with sex for any tract (FDR q’s < 0.05).

### 3.6 Supplemental analyses

A supplemental analysis examined the additional variance accounted for by pubertal hormones over and above age and sex without pubertal stage in the model. These results follow similar patterns to the main analysis with a few differences. Six tracts had different best-fitting models. The best fit model for the corpus callosum prefrontal body (DHEA in main text), genu (DHEA in main text), and the inferior longitudinal fasciculus (Estradiol in main text) was the Pubertal Timing model. The best fit model for the corpus callosum premotor body (Age in main text), and Frontal Aslant Tract (Age in main text) was the DHEA model. The splenium (DHEA in main text) and acoustic radiation (Age in main text) were the best fit by the Estradiol model. Thus, some hormone models are the best fit models when pubertal status is not included. This suggests a potential role of those hormones in relation to FA within those tracts but not above and beyond what is accounted for by pubertal status, which was the criteria that was used in the main text analyses. Complete results and brief discussion are provided in the Supplemental Figure S4.

A second supplemental analysis was conducted in which pubertal variables were given priority over age in the sequential modeling with age added last (as part of the full model). Results followed similar patterns as the main text results, suggesting age is an important factor explaining FA in most tracts. The full model that includes age was the best fit for all but one tract, which was the middle cerebellar peduncle. Complete results and brief discussion are provided in the Supplemental Figure S5.

A third supplemental analysis was conducted using a restricted age range of 9–18-year-olds (N = 662) that captures the age window of just active pubertal development captured by the pubertal stages. There were some interesting differences within this age range. Age accounted for less unique additional variance and pubertal terms accounted for slightly more additional variance in most tracts (Supplemental Fig. S6). The ventral cingulum bundle and extreme capsule that were best fit by Estradiol in the main text, were best fit by the Age model. This could be due to the nonlinear positive associations between FA and age within these tracts, particularly in the younger individuals who were excluded from this analysis. The variation in estradiol levels during this early time period could be related to this nonlinear increase in FA. In addition, age accounted for less unique variance, while pubertal stage accounted for slightly more in almost all tracts compared to the full sample. Thus, this analysis highlights that different pubertal variables might have more of an influence on white matter microstructure in an age range that is restricted to a more active pubertal development.

Finally, a sensitivity analysis was conducted excluding individuals using hormonal birth control (N = 59). Excluding individuals on birth control slightly changed the best-fitting model for four tracts. The superior longitudinal fasciculus 1 was best fit by the Age model (Pubertal Timing in main text). The genu was best explained by the Pubertal Timing model (DHEA model in main text). The acoustic radiation (Age in main text) and uncinate fasciculus (Estradiol in main text) were best fit by the full model. The additional variance explained by each model remained similar to the results from the main text (Supplemental Fig. S7). The sample of individuals on hormonal contraceptives was too small to perform meaningful analyses that fit in the scope of this study. Future studies can explicitly study these potential effects of hormonal birth control on white matter microstructure. Complete results and brief discussion are provided in the Supplementary Data.

## 4 Discussion

This work examined how pubertal factors are uniquely related to white matter microstructure during adolescence. FA within the majority of white matter tracts examined had nonlinear relationships with age. Sex and age accounted for the most unique FA variance in most tracts. While most tracts were best fit by Age models, the pubertal timing model was the best fit for the inferior longitudinal fasciculus. Out of the four hormones examined, DHEA and estradiol were the hormones that seemed to be more related to white matter microstructure over and above age and pubertal stage. RD explained much more variance in FA than AD. Finally, the FA variance explained by Pubertal Timing and DHEA were significantly positively associated with the S-A axis, demonstrating that Pubertal Timing and DHEA explain more FA variance in association-related tracts, rather than sensorimotor-related tracts.

White matter in the brain undergoes many subtle changes during adolescence. These results show that FA is positively associated with age in most canonical white matter tracts, suggesting underlying microstructure changes in the tracts. In this large, cross-sectional sample, most tracts appear to undergo nonlinear increases in FA during the younger ages (~5–12). In addition, the Age model was the best-fitting model for the majority of white matter tracts, and age explained the most unique variance in FA in almost every tract. Age-related increases in FA during adolescence has also previously been suggested in both cross-sectional and longitudinal data in smaller samples and narrower age ranges (Herting et al., 2012, 2017; Ho et al., 2020; Lebel & Beaulieu, 2011). In addition to age, sex explained a relatively large amount of unique variance in FA in most tracts. Sex differences in FA have been previously reported (Lebel et al., 2010; Piekarski et al., 2023; Schmithorst et al., 2008). Here, the unique variance contributed by sex is explained by a main effect of sex, since there were no significant interactions with sex in relationships between age and FA. While there were no significant interactions with sex, the variability in hormone levels differ between females and males (Supplemental Fig. S1). When one group has a restricted range for specific hormones, the sample may be underpowered to detect true interactions. The nonlinear increase in FA experienced by both males and females in most tracts occurs around the time of pubertal onset, which is a key period of increased hormonal productions and other psychosocial events that likely contribute to FA changes.

Here, we teased apart some of the unique relationships that these pubertal mechanisms have with these microstructural changes. Pubertal timing was the best-fitting model for the superior longitudinal fasciculus I (SLF1). The pubertal timing definition utilized here was the degree to which an individual’s puberty deviates from the average of same aged peers. While other definitions exist (e.g., age of menarche), this is the commonly used definition of pubertal timing and what we used previously in our investigation of gray matter structural changes in a largely overlapping sample. Since this tract experiences a significant increase in FA from ages 6–12 years old, which corresponds to the onset of puberty in many individuals, differences in pubertal timing could have a larger influence on this higher-level motor related tract. Differences in pubertal timing can represent differences in direct effects of pubertal hormones or indirect effects from changes in psychosocial processes, such as peer relations and social cognition that may also shape brain development (Barendse et al., 2022; McLaughlin et al., 2019; Pfeifer & Allen, 2021). Changes in puberty may also interact with age-related changes. For example, completing puberty may decelerate age-related FA trajectories. Future work can directly examine these potential complex interactions and how different psychosocial factors may potentially impact pubertal timing and the relationship with white matter microstructure in the SLF1.

The DHEA and estradiol models were the best fit for FA within certain white matter tracts. Greater DHEA levels (adjusted for age and pubertal stage) were related to lower levels of FA in the anterior and posterior corpus callosum, while greater estradiol levels were related to less FA in more inferior association tracts. In addition, the full model with all hormones was the best fit for FA in the rostrum. Previous studies showed mixed results on how pubertal hormones were related to FA, but greater DHEA levels had previously been shown to be associated with higher corpus callosum RD (Barendse et al., 2018). Since RD and FA are typically inversely related, these results are consistent and suggest that relatively higher levels of DHEA may be related to less structural integrity in the corpus callosum. Similarly, it was previously shown that age-adjusted Estradiol levels were negatively related to FA in parietal regions and inferior fronto-occipital fasciculus (Herting et al., 2012; Stoica et al., 2019). In the context of overall positive relationships between FA and age, this suggests that increased levels of these hormones relative to peers of similar age and pubertal status are related to lower levels of FA, potentially indicating relatively slower white matter microstructure maturation*.* DHEA and estradiol have been shown to impact myelination mechanisms and increase glial plasticity (Long et al., 2021; Maninger et al., 2009; Nguyen et al., 2016).

We found that variation in FA was explained much more by radial diffusivity than axial diffusivity. Both RD and AD can impact the degree of anisotropy measured by FA. The positive association between FA and age could be due to a number of potential mechanisms such as the thickening of axons from myelination, increase in fiber density within the tract, or increase in diameter of the axons, all ultimately increasing diffusion anisotropy and thereby increasing FA. It was previously suggested that FA changes during adolescent development were explained by changes in RD, though some had suggested an influence from both RD and AD (Ashtari et al., 2007; Faria et al., 2010; Giorgio et al., 2008; Lebel & Beaulieu, 2011). Here in a large sample, we found that RD explains considerably more FA variance than AD during adolescence. While early literature frequently positioned RD as an indicator of myelination (e.g., Kumar et al., 2014; Lebel et al., 2012; Madden et al., 2012; Song et al., 2003), more recent literature shows that RD is not purely sensitive to myelin content. These developmental microstructural changes can be related to axonal packing, axon diameter, and myelin (Genc et al., 2023; Lebel & Deoni, 2018; Paus & Toro, 2009; Pesaresi et al., 2015). Thus, caution is warranted in interpreting the RD changes without confirmation from more invasive or sensitive methods to delineate specific underlying mechanisms (see limitations below) (Paus, 2010).

Finally, the FA variance explained by Pubertal Timing and DHEA was positively related to the S-A axis, suggesting these explain more FA variance in association-related tracts than sensorimotor related tracts. This aligns with the previous literature that suggests associations between pubertal hormones and cortical variability in humans and animal models are most commonly found in association cortex (Sydnor et al., 2021). Thus, Pubertal Timing and DHEA, which continue to increase throughout adolescence, could have a greater impact on the white matter tracts with endpoints in association-related cortices and experience more protracted developmental trajectories, potentially allowing for a longer period of hormonal impact.

While this study has many strengths, including a large sample size and inclusion of multiple puberty-related features, some limitations need to be considered. This was a cross-sectional study, and thus, we were unable to examine these relationships within individuals during their development. Since a subsample of these individuals were followed for two additional timepoints during adolescence, future studies can analyze intra-individual longitudinal changes. The effects of pubertal tempo (the speed of change in physical pubertal development), which has also been suggested to be associated with structural brain development (Chahal et al., 2018; Herting et al., 2017; Ho et al., 2020), can also be addressed in the longitudinal sample. Further, although this study included a large sample size, the questions addressed remain relatively understudied and the findings would benefit from replication.

With the wide age-range it is important to consider that cyclical variations in estradiol and progesterone become more pronounced in older adolescent females. To account for this, saliva sampling was measured at a time of naturally low levels in postmenarcheal females with regular cycles (cycle day 7) to facilitate their analysis with premenarcheal females, females with irregular cycles, and males. However, this approach did not allow for hormonal peaks to be characterized. Hormones can also be influenced by circadian and sleep-wake cycles, and while participants were instructed to collect samples within 30 minutes of waking, this dataset only collected information on when participants collected the sample, which was generally early in the morning (mean time of 8:16 am), and did not collect the time they awoke. Finally, other factors may influence hormone levels and brain development (e.g., physical activity, substance use). Overall, a single saliva data collection may not capture an individual’s typical, average, or cumulative hormone level. This unmodelled within-subject hormonal variability might impact the observed results. Future studies could include repeated hormone sampling within individuals to examine these relationships in more detail.

This analysis focused on FA, as it is sensitive to microstructural changes and is the most commonly used metric for quantifying diffusion MRI, and thus relevant to a large volume of other literature. Further, with the high-quality data in a large dataset, this study teased apart the relative contributions of radial and axial diffusivity to FA. However, future analyses could include mean diffusivity (the average of the tensor eigenvalues) to investigate these relationships. This analysis included multishell diffusion data, including high b-values in fitting the basic diffusion tensor model. Notably, high b-value data violates the assumption of the DTI model of Gaussian diffusion with simple mono-exponential decay. Pines et al. (2020) empirically demonstrated that DTI measures (FA, RD, and AD) calculated from multi-shell data with a high maximum b-value (b = 300, 800, and 2000 s/mm^2^) are, nonetheless, highly correlated with values obtained by using just the b = 800 shell (r’s > 0.9) and showed similar developmental trajectories (Pines et al., 2020). The estimates of FA, RD, and AD in this study will be biased to some degree relative to what would be obtained in a DTI model using lower b-value data. However, the relationships between the DTI measures employed here (FA, RD, and AD) with other factors (e.g., age, puberty, and hormones) are valid. Nonetheless, future studies could benefit by fitting FA via Diffusion Kurtosis Imaging (DKI) to generate more accurate FA values themselves. Additionally, models such as NODDI (Zhang et al., 2012) could be used to evaluate other measures of white matter microstructure as well. Finally, while this study included high-quality DWI data, the measures of FA, RD, and AD are indirect measurements of underlying microstructure, and lack the specificity to distinguish the multiple underlying axon and myelin-related processes in neurodevelopment. Thus, caution is needed when interpreting potential underlying mechanisms without the addition of more sensitive or ex vivo methods.

This work found that sex and age accounted for the most unique FA variance in most tracts during adolescence. Pubertal timing was related to the SLF1, and out of the four hormones examined, DHEA and estradiol seemed to be most related to white matter microstructure. Pubertal Timing and DHEA were more related to FA in association-related tracts than sensorimotor tracts. Thus, the elements of puberty captured by pubertal timing and DHEA are more uniquely related to white matter microstructure, in tracts related to later maturing association cortices*.* As these patterns exist in typically developing individuals, alterations in these pubertal mechanisms (timing and hormones) and the patterns of their relationships with microstructural development may also play a role in risk for psychopathology during this particularly vulnerable time of adolescence.

## Supplementary Material

Supplementary Material

## Data Availability

The HCP-Development Lifespan 2.0 Release is available for download from the NIMH Data Archive (NDA). Information about the release is available at: https://www.humanconnectome.org/study/hcp-lifespan-development/data-releases. The 2.0 Release includes cross-sectional visit 1 (V1) preprocessed structural and functional imaging data, unprocessed V1 imaging data for all included modalities (structural, resting-state fMRI, task fMRI, diffusion, and ASL), and non-imaging demographic and behavioral assessment data from 652 HCP-Development (HCP-D, ages 5–21) healthy participants. This manuscript used cross-sectional data from 1301 HCP-D participants, many of whom are not in the 2.0 Release, but will be available in the 3.0 Release.
