# Supplementary material for: Disentangling the unique associations of age, pubertal stage, and pubertal hormones with white matter microstructure in childhood and adolescence
Source: Imaging Neurosci (Camb). 2026 Jun 15;4:IMAG.a.1270. doi: 10.1162/IMAG.a.1270 (PMC13271154; doi:10.1162/IMAG.a.1270)
Supplement: Supplementary Material [file IMAG.a.1270_supp.pdf]

Supporting Information for “Disentangling the Unique Associations of Age, Pubertal Stage, and Pubertal Hormones with White Matter Microstructure in Childhood and Adolescence”

Authors: Mark Curtis[a*], Anastasia Yendiki[b], Theresa Cheng [c], Adam Omary[c], John C. Flournoy[c], Sridhar Kandala[d], Ashley F. P. Sanders[d], Michael P. Harms[d], Leah H. Somerville[b], and Deanna M. Barch[a]

Affiliations:
[a] Department of Psychological and Brain Sciences, Washington University in St. Louis, MO 63110, USA
[b] Athinoula A. Martinos Center for Biomedical Imaging, Massachusetts General Hospital and Harvard Medical School, Boston, MA
[c] Department of Psychology and Center for Brain Science, Harvard University, Cambridge, MA 02138, USA
[d] Department of Psychiatry, Washington University School of Medicine, St. Louis, MO 63110, USA
*Corresponding author. Mark Curtis

Mark Curtis
Email: mark.c@wustl.edu

This PDF file includes:

Supporting text
Supporting Results and Brief Discussion
Tables S1 to S4
Figures S1 to S7
SI References

Supporting Information Text

Supplemental Results: Age-adjusted Hormone Models (without giving priority to pubertal stage)

It was also of interest to examine if any pubertal variable added additional variance above and beyond age when each was examined individually. To accomplish this, a supplemental analysis was performed that treated pubertal stage and hormones similarly by adding them individually to the Age model, instead of including pubertal stage in the hormone models. The following are the differences from the main results for this additional analysis.

The best fit model for the corpus callosum prefrontal body (DHEA in main text), genu (DHEA in main text), and the inferior longitudinal fasciculus (Estradiol in main text) was the Pubertal Timing model. The best fit model for the corpus callosum premotor body (Age in main text), and Frontal Aslant Tract (Age in main text) was the DHEA model. The splenium (DHEA in main text) and acoustic radiation (Age in main text) were best fit by the Estradiol model. When not including pubertal status in the hormone models, hormones still accounted for a relatively small amount of additional variance above and beyond sex and age in most tracts (**Figure S4**).

Age-adjusted Hormone Models Brief Discussion

In this analysis, some hormone models are the best fit models (meaning that they account for meaningful additional variance, not that they account for the most variance) when pubertal status is not included. This suggests a potential role of those hormones in relation to FA within those tracts. However, it does not mean those hormones account for significantly more variance above and beyond what is accounted for by pubertal status, which was the criteria that was used in the main text analyses. In addition, the corpus callosum prefrontal body and genu were best fit by the DHEA model in the main text but best fit by the Pubertal Timing model in this analysis. This suggests that pubertal status explains more additional variance above age and sex than DHEA, but DHEA explains meaningful additional variance above sex, age, and pubertal status.

Supplemental Results: Puberty-Adjusted Age Models

A supplemental analysis was done in which the sequential addition of pubertal variables to the models did not include age. The final full model did include age. This aimed to investigate the relative contributions of pubertal variables without age in the models and a full model that included all pubertal variables and age. The following are the differences from the results from the main text.

With this ordering of the sequential modeling, the full model that included pubertal variables and age was the best fitting model for FA in all but 1 tract, which was the middle cerebellar peduncle. The middle cerebellar peduncle was best described by the Sex model, which was the same as the main text. This indicates that for most of the tracts, age is a critical variable that accounts for meaningful variance over and above the other variables (as determined by the AIC criterion), even when included last in the sequential modeling. The additional R^2 analysis showed similar results as the main text (where age was added first) for how much explained variance the hormones added, even though they were added prior to age (**Figure S5**). However, here pubertal stage explains more additional variance in most tracts compared to the baseline sex model,

which is to be expected since pubertal stage and age are highly correlated, and age is no longer in the pubertal stage model.

Puberty-Adjusted Age Models Brief Discussion

This supplemental analysis aimed to examine how pubertal stage and hormones contribute to structure when age is not given priority in the sequential modeling. FA in most tracts were best fit by the full model that included age. This analysis demonstrates that age still explains meaningful variance above and beyond the pubertal variables.

Supplemental Results: Restricted Age Range (9-18 years old)

A supplemental sensitivity analysis was done in a restricted age range of 9–18-year-olds (N=662) that captures the age window of active development of puberty as indexed by the pubertal stages. The following are the differences in results compared to the full sample.

In this age range, FA in the corticospinal tract (Age in main text) were best fit by the Sex model. The ventral cingulum bundle (Estradiol in main text), extreme capsule (Estradiol in main text), SLF1 (Pubertal Timing in main text), and middle cerebellar peduncle (Sex in main text) were best fit by the Age model. The inferior longitudinal fasciculus (Estradiol in main text), genu (DHEA in main text), splenium (DHEA in main text), optic radiation (Age in main text) and fornix (Age in main text) were best fit by the Pubertal Timing model. FA in the rostrum (Full model in main text) was best fit by Estradiol model. The central body of the corpus callosum was best fit by the progesterone model (Age model in main text). The acoustic radiation was best fit by the full model (Age in the main text). Age accounted for less additional variance in most tracts compared to the full sample (**Figure S6**).

Restricted Age Range Brief Discussion

There were interesting differences within this restricted age range. The ventral cingulum bundle and extreme capsule that were best fit by Estradiol in the main text, were best fit by the Age model. This could be due to the significant nonlinear age-related increases in FA within these tracts in the younger individuals who were excluded from this analysis. The variation in estradiol levels during this early time period could be related to this nonlinear increase in FA. In addition, age accounted for less unique variance, while pubertal stage accounted for slightly more in almost all regions compared to the full sample. Thus, this analysis highlights that different pubertal variables might have more (or less) of an influence on white matter microstructure in an age range that is restricted to a range of more active pubertal development.

Supplemental Results: Birth Control Analysis

Fifty-nine individuals reported taking hormonal birth control. Since birth control influences hormone levels, an analysis was performed in which those taking birth control were removed to investigate the potential influence on the results (N=1046). The results remained the same other than the following differences.

The superior longitudinal fasciculus 1 was best fit by the Age model (Pubertal Timing in main text). The genu was best explained by the Pubertal Timing model (DHEA model in main text). The acoustic radiation (Age in main text) and uncinate fasciculus (Estradiol in main text) were best fit by the full model. The additional variance explained by each model remained similar to the results from the main text (**Figure S8**).

Birth Control Brief Discussion

Excluding individuals on birth control slightly changed the best fitting model for three tracts. The focus of this study was to examine these relationships in a relatively normative sample, which would include some individuals taking birth control. In addition, there was not a large enough sample of individuals on hormonal contraceptives to perform meaningful analyses that fit in the scope of this study. Future studies can explicitly study these potential effects of hormonal birth control on white matter microstructure.

Supplemental Materials and Methods

Participants

Inclusion criteria included the following: (i) age 5-21 years; (ii) speaks English well; and (iii) safe to collect MRI. Exclusion criteria for recruitment included the following: (i) premature birth (>3 weeks early, or weighed <5 pounds, or neonatal ICU stay >2 days); (ii) any history of serious medical conditions (e.g., stroke, cerebral palsy); (iii) serious endocrine conditions (e.g., precocious puberty, untreated growth hormone deficiency); (iv) long term use of immunosuppressants or steroids; (v) any history of serious head injury; (vi) any hospitalization >2 days for certain physical or psychiatric conditions or substance use; (vii) treatment >12 months for psychiatric conditions; (viii) receiving certain special services at school (e.g., dyslexia or other learning disability); (ix) claustrophobia; or (x) pregnancy.

Pubertal Stage Scales

The Morris-Udry scale (Morris & Udry, 1980) and the Pubertal Development Scale (PDS) were used to measure self-reported physical markers of development and secondary sex characteristics (Petersen et al., 1988). For the Morris-Udry scale, participants identified 1 of 5 images that best represented pubic hair development and either breast development in females or genitalia development in males. For the PDS, participants answered questions on growth spurt, body hair, and skin changes. In addition, males responded to questions about voice changes and facial hair, while females responded to questions about breast development and menarche. Participants rated each question on the following four-point scale: (1) has not begun, (2) has barely begun, (3) has definitely begun, and (4) appears complete. The question to females about menarche consisted of a yes/no answer (no=1, yes=4). The PDS scores were transformed based on Shirtcliff and colleagues (2009) (Shirtcliff et al., 2009), where the scores were adjusted to a 5-point scale to parallel the five Tanner stages, the standard system for describing the physical changes associated with pubertal development.

Parents completed these questionnaires on behalf of children aged 5–13 years old. Participants aged 9–21 years completed the PDS on their own. Since child and parent responses were strongly correlated for the Morris-Udry ($r=0.78$) and the PDS ($r=0.79$), the children's self-report scores were utilized for those aged 9 and older, with parent report used for ages 5–8 years. The scores from the individual questions were averaged for both Morris-Udry and PDS and then converted to z-scores. The z-scored data were highly correlated between the two scales ($r=0.89$), and thus the two z-scores were averaged to calculate a singular pubertal composite score for each individual (Curtis et al., 2024).

Hormone Acquisition and Processing

The assay sensitivity limits for each hormone were as follows: DHEA: 10.2-1000 pg/ml, Testosterone: 6.1-600 pg/ml, Estradiol: 1-32 pg/ml, Progesterone: 10-2430 pg/ml. There were three extreme outliers that were excluded as it was unlikely these were biologically plausible hormone values (>2.5 times the upper sensitivity limit). All other data outside of these sensitivity limits were handled as follows: if the assay produced an undetectable value below the lower sensitivity threshold, the hormone value was set to 0 (DHEA: N=28, Testosterone: N=29, Estradiol: N=67, Progesterone: N=8) (Herting et al., 2020). If the value was below the lower sensitivity threshold but detectable, the original value was retained. Finally, values above the upper sensitivity threshold were winsorized to be equal to the threshold value (N=9). These decisions were made to maximize the amount of data utilized and not bias the data by excluding biologically plausible hormone values, while not allowing extreme outliers to have undue influence on the models.

Image Acquisition & Processing Quality Control

To improve data acquisition quality, the acquisition included volumetric navigators to detect motion and motion corrupted k-space lines were selectively reacquired (Harms et al., 2018; Tisdall et al., 2016). Scans were reacquired in their entirety if deemed to be of poor quality. Only the single pair of T1w and T2w scans of highest quality were utilized. Scan reconstructed with Siemens 'PreScan Normalize' feature (to reduce the receive coil bias field) were used to review scan quality at the scanner, but non-normalized reconstructions were used as inputs for processing (a choice made by HCP to maintain consistency with the processing of the HCP Young-Adult data).

The MRI data were analyzed using the HCP Pipelines (v4.0.0 or later; all versions involved the same structural processing) (Glasser et al., 2013) instantiated into the QuNex container environment (qunex.yale.edu), using the same processing as the Lifespan HCP Release 2.0 in the NIMH Data Archive (nda.nih.gov). Briefly, T1w and T2w volumes were processed through the PreFreeSurfer pipeline, which included gradient nonlinearity distortion correction, registration of the T2w volume to the T1w volume, bias field correction, brain-extraction, and AC-PC alignment in native space. The 'white' and 'pial' surfaces were computed in FreeSurfer (v6.0.0) (Dale et al., 1999; Fischl & Dale, 2000) and were refined with the T2w data. The PostFreeSurfer pipeline produced cortical surfaces in GIFTI format and surface-related data in CIFTI format, with each subject's cortical surface registered to a common mesh ('32k_FS_LR') using 'MSMAll' reg, an areal-feature-based cortical surface registration which utilizes cortical myelin maps and resting-state network maps (Glasser et al., 2016; Robinson et al., 2014). Previous review of the HCP-D data revealed artifacts in the longer echoes of the MPRAGE acquisition, leading to the use of the mean of the images derived from the shortest two echoes (i.e., excluding the longest two of four echoes) as the T1w input to the HCP Pipelines (Elam et al., 2021). After processing through the PreFreeSurfer, FreeSurfer and PostFreeSurfer pipelines as described in the main text, an experienced individual performed a manual 'SurfaceQC' review of the white and pial surface placement, which included inspection for dural inclusions and incomplete capture of the cortical ribbon. T1w/T2w ratio "myelin maps" were also examined, which are sensitive to errors in the surface placement and thus useful for efficient QC of the surfaces (Elam et al., 2021; Glasser & Van Essen, 2011).

Missing Data

Out of 1,264 individuals with quality MRI data, four individuals had poor TRACULA results in at least 1 tract, three individuals had missing hormone and puberty data, 14 were missing puberty data, 83 were missing all hormone data, 4 were missing

DHEA values only, 4 were missing testosterone only, 7 were missing estradiol only, 2 were missing progesterone only, and 9 were missing two or three hormones. There was one individual with estradiol and two individuals with progesterone values that were extreme outliers and thus excluded (see above). An additional 26 individuals were excluded for having excessive motion (see main text for criteria), leaving a total of 1,105 participants with complete data (12.6% participants excluded).

Eddy Parameters
These are the specific ‘eddy’ parameters used: niter=8, fwhm=’10,8,6,4,2,0,0,0,0’, nvoxhp=2000, repol, ol_type=both, ol_nstd=5, with_outliers, mporder=16, s2v_niter=8, estimate_move_by_susceptibility, mbs_niter=20, initrand, residuals.

Supplemental Tables:

Supplemental Table 1. Spearman correlations between age, pubertal stage, and hormones. All correlations in the full sample were significant after correcting for multiple comparisons (p<0.0033 uncorrected). Correlations are also presented separately for females and males.

Measure	1	2	3	4	5
**Full Sample**					
**1. Age**	—				
**2. Pubertal Composite**	0.89	—			
**3. DHEA**	0.76	0.73	—		
**4.Testosterone**	0.70	0.63	0.70	—	
**5. Estradiol**	0.50	0.49	0.68	0.43	—
**6. Progesterone**	0.15	0.13	0.40	0.20	0.46
**Females**					
**1. Age**	—				
**2. Pubertal Composite**	0.90	—			
**3. DHEA**	0.76	0.73	—		
**4.Testosterone**	0.59	0.58	0.68	—	
**5. Estradiol**	0.56	0.54	0.70	0.47	—
**6. Progesterone**	0.17	0.14	0.38	0.18	0.45
**Males**					
**1. Age**	—				
**2. Pubertal Composite**	0.91	—			
**3. DHEA**	0.76	0.73	—		
**4.Testosterone**	0.83	0.82	0.80	—	
**5. Estradiol**	0.44	0.42	0.66	0.50	—
**6. Progesterone**	0.13	0.13	0.43	0.23	0.48

Supplemental Table 2. Generalized additive model statistical outputs for the relationship between age and FA for each tract. Most tracts exhibited nonlinear FA-age relationships.

Tract	EDF	F-value	P-Value
Acomm	2.94	37.10	<2e-16
cc.bodyc	3.68	43.17	<2e-16
cc.bodyp	3.12	40.54	<2e-16
cc.bodypf*	1.85	24.97	<2e-16
cc.bodypm*	1.01	267.20	<2e-16
cc.bodyt	2.70	45.85	<2e-16
cc.genu*	1.01	6.66	0.009
cc.rostrum*	1.01	3.23	0.072
cc.splenium	3.48	13.80	<2e-16
AF	3.33	38.31	<2e-16
AR	3.70	64.44	<2e-16
ATR	3.77	37.31	<2e-16
CBD	3.14	57.77	<2e-16
CBV	3.37	46.06	<2e-16
CST	5.14	19.30	<2e-16
EMC	3.50	50.78	<2e-16
FAT	4.14	44.35	<2e-16
FX	2.59	58.46	<2e-16
ILF	2.95	18.34	<2e-16
MLF	3.70	73.72	<2e-16
OR	3.12	14.37	<2e-16
SLF1	4.21	65.11	<2e-16
SLF2	3.56	39.76	<2e-16
SLF3	3.86	24.22	<2e-16
UF	3.29	13.89	<2e-16
MCP*	1.62	0.521	0.593

* denotes linear relationship

Tracts: Acomm, Anterior Commissure; CC, Corpus Callosum, which has the following sub-parts: Bodyc, Central Body; Bodyp, Parietal Body; Bodypf, Prefrontal body; Bodypm, Premotor body; Bodyt, Temporal Body; Genu; Rostrum; Splenium; AF, Arcuate Fasciculus; AR, Acoustic Radiation; ATR, Anterior Thalamic Radiation; CBD, Dorsal Cingulum Bundle; CBV, Ventral Cingulum Bundle; CST, Corticospinal Tract; EMC, Extreme Capsule; FAT, Frontal Aslant Tract; FX, Fornix; ILF, Inferior Longitudinal Fasciculus; MLF, Middle Longitudinal Fasciculus; OR, Optic Radiation; SLF, Superior Longitudinal Fasciculus; UF, Uncinate Fasciculus; MCP, Middle Cerebellar Peduncle

Supplemental Table 3. FA summary statistics for each tract.

Tract	Mean±SD	Range (Min-Max)
Acomm	0.42±0.02	0.33-0.51
cc.bodyc	0.60±0.03	0.48-0.71
cc.bodyp	0.60±0.03	0.46-0.68
cc.bodypf	0.59±0.03	0.51-0.68
cc.bodypm	0.62±0.03	0.53-0.70
cc.bodyt	0.57±0.02	0.44-0.64
cc.genu	0.59±0.03	0.50-0.67
cc.rostrum	0.59±0.03	0.49-0.70
cc.splenium	0.68±0.03	0.50-0.76
AF	0.51±0.02	0.42-0.57
AR	0.52±0.02	0.41-0.59
ATR	0.51±0.02	0.42-0.61
CBD	0.61±0.03	0.48-0.71
CBV	0.56±0.03	0.43-0.64
CST	0.55±0.02	0.47-0.61
EMC	0.52±0.03	0.41-0.60
FAT	0.53±0.02	0.43-0.60
FX	0.44±0.02	0.37-0.50
ILF	0.57±0.02	0.48-0.64
MLF	0.54±0.03	0.44-0.61
OR	0.57±0.02	0.48-0.64
SLF1	0.57±0.02	0.48-0.64
SLF2	0.47±0.02	0.38-0.54
SLF3	0.49±0.02	0.40-0.56
UF	0.51±0.02	0.43-0.57
MCP	0.52±0.02	0.44-0.59

Tracts: Acomm, Anterior Commissure; CC, Corpus Callosum, which has the following sub-parts: Bodyc, Central Body; Bodyp, Parietal Body; Bodypf, Prefrontal body; Bodypm, Premotor body; Bodyt, Temporal Body; Genu; Rostrum; Splenium; AF, Arcuate Fasciculus; AR, Acoustic Radiation; ATR, Anterior Thalamic Radiation; CBD, Dorsal Cingulum Bundle; CBV, Ventral Cingulum Bundle; CST, Corticospinal Tract; EMC, Extreme Capsule; FAT, Frontal Aslant Tract; FX, Fornix; ILF, Inferior Longitudinal Fasciculus; MLF, Middle Longitudinal Fasciculus; OR, Optic Radiation; SLF, Superior Longitudinal Fasciculus; UF, Uncinate Fasciculus; MCP, Middle Cerebellar Peduncle

Supplemental Table 4. The sensorimotor-association axis value and rank order for each white matter tract.

Tract	S-A Rank Value	Tract S-A Rank Order
cc.bodyc	8204	1
OR	10673	2
cc.splenium	13159	3
CST	16429	4
cc.bodyp	22268	5
CBV	26787	6
MLF	26995	7
ILF	27150	8
AR	27205	9
cc.bodyt	35028	10
SLF1	36578	11
CBD	37547	12
cc.bodypm	37946	13
SLF3	38612	14
FAT	41210	15
FX	41345	16
SLF2	42152	17
acomm.1	42830	18
AF	43155	19
UF	46773	20
cc.rostrum	50541	21
EMC	51353	22
ATR	52337	23
cc.genu	53064	24
cc.bodypf	53902	25

Tracts: Acomm, Anterior Commissure; CC, Corpus Callosum, which has the following sub-parts: Bodyc, Central Body; Bodyp, Parietal Body; Bodypf, Prefrontal body; Bodypm, Premotor body; Bodyt, Temporal Body; Genu; Rostrum; Splenium; AF, Arcuate Fasciculus; AR, Acoustic Radiation; ATR, Anterior Thalamic Radiation; CBD, Dorsal Cingulum Bundle; CBV, Ventral Cingulum Bundle; CST, Corticospinal Tract; EMC, Extreme Capsule; FAT, Frontal Aslant Tract; FX, Fornix; ILF, Inferior Longitudinal Fasciculus; MLF, Middle Longitudinal Fasciculus; OR, Optic Radiation; SLF, Superior Longitudinal Fasciculus; UF, Uncinate Fasciculus; MCP, Middle Cerebellar Peduncle

Supplemental Figures

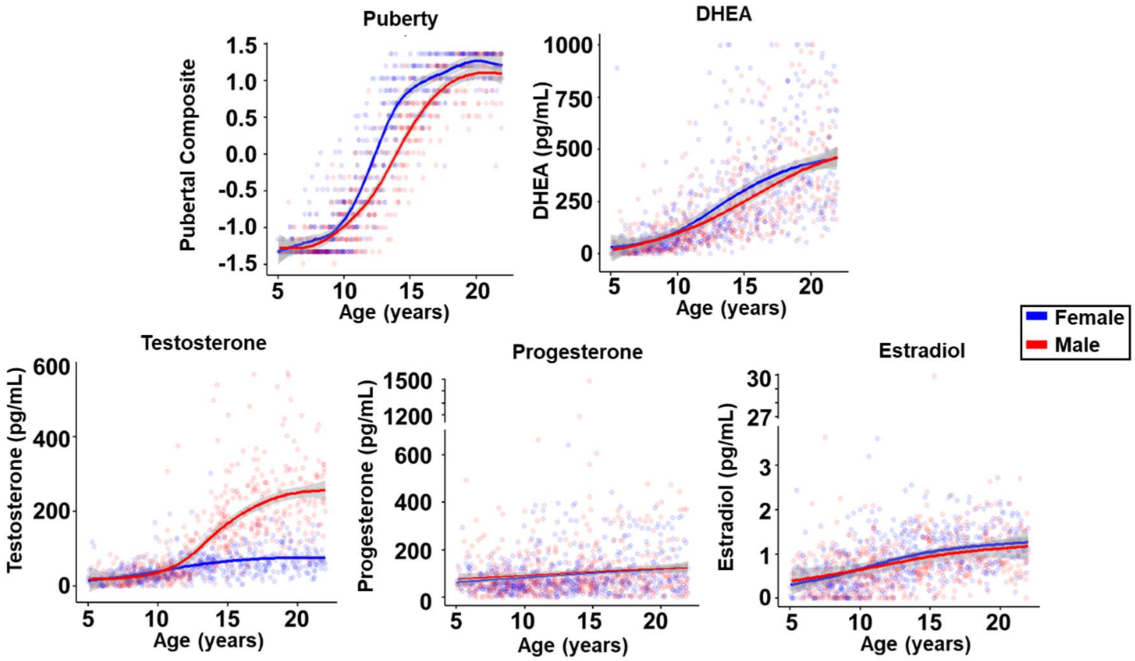

Supplemental Figure 1. Age-related changes in pubertal stage and hormone levels. These are the age-related trajectories of the pubertal variables from the participants included in this study (N=1,105). See Omary and colleagues (2025) for a comprehensive analysis of pubertal and hormonal data from an expanded HCPD sample (Omary et al., 2025).

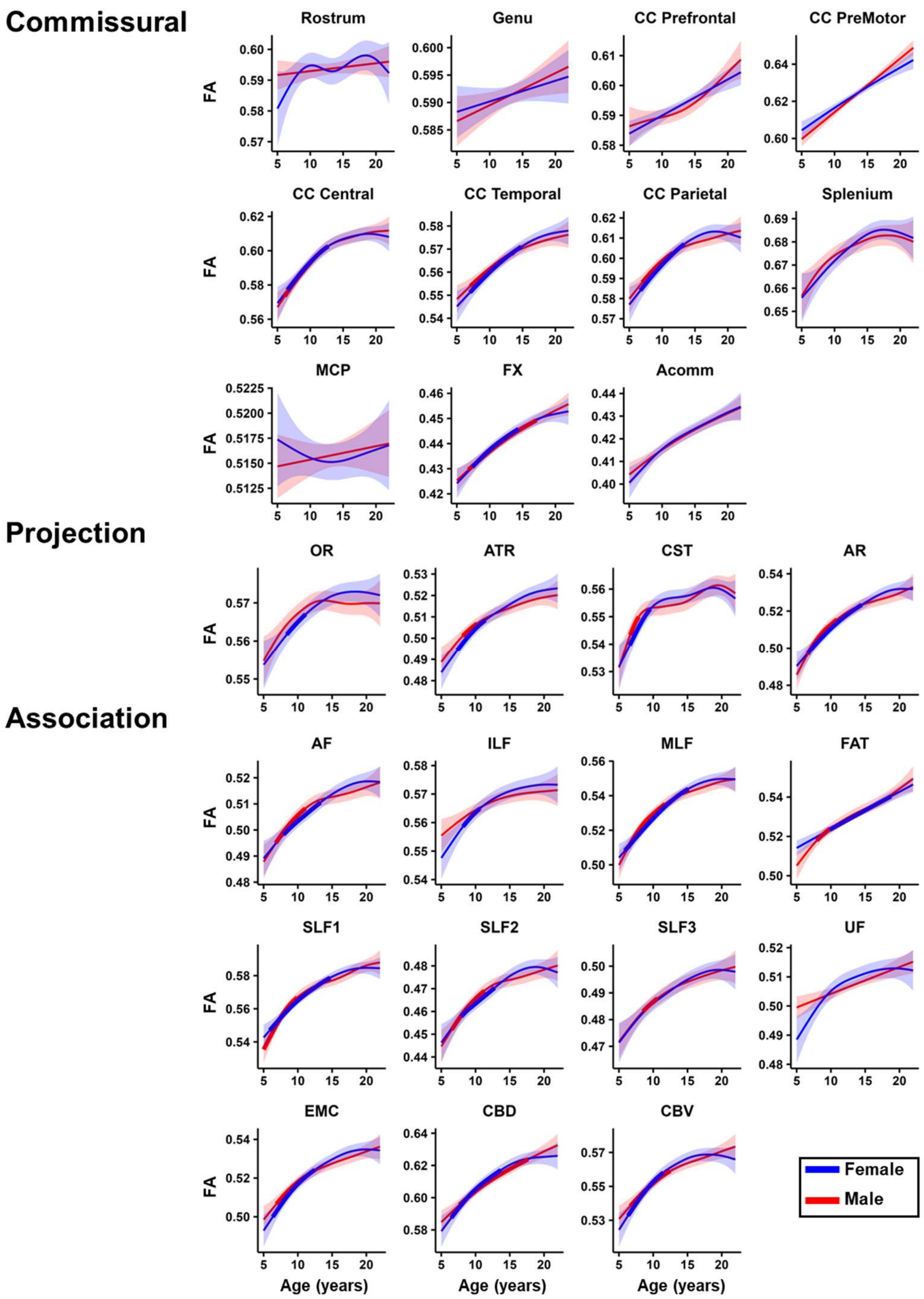

Supplemental Figure 2. Age-related FA Changes. The same age-related changes for FA in each canonical tract presented in Figure 1 with the y-axis range customized for each tract to highlight the trajectory shape. See Figure 1 for more details.

Tracts**:** Acomm, Anterior Commissure; CC, Corpus Callosum, which has the following sub-parts: Central Body; Parietal Body; Prefrontal body; Premotor body; Temporal Body; Genu; Rostrum; Splenium; AF, Arcuate Fasciculus; AR, Acoustic Radiation; ATR, Anterior Thalamic Radiation; CBD, Dorsal Cingulum Bundle; CBV, Ventral Cingulum Bundle; CST, Corticospinal Tract; EMC, Extreme Capsule; FAT, Frontal Aslant Tract; FX, Fornix; ILF, Inferior Longitudinal Fasciculus; MLF, Middle Longitudinal Fasciculus; OR, Optic Radiation; SLF, Superior Longitudinal Fasciculus; UF, Uncinate Fasciculus; MCP, Middle Cerebellar Peduncle

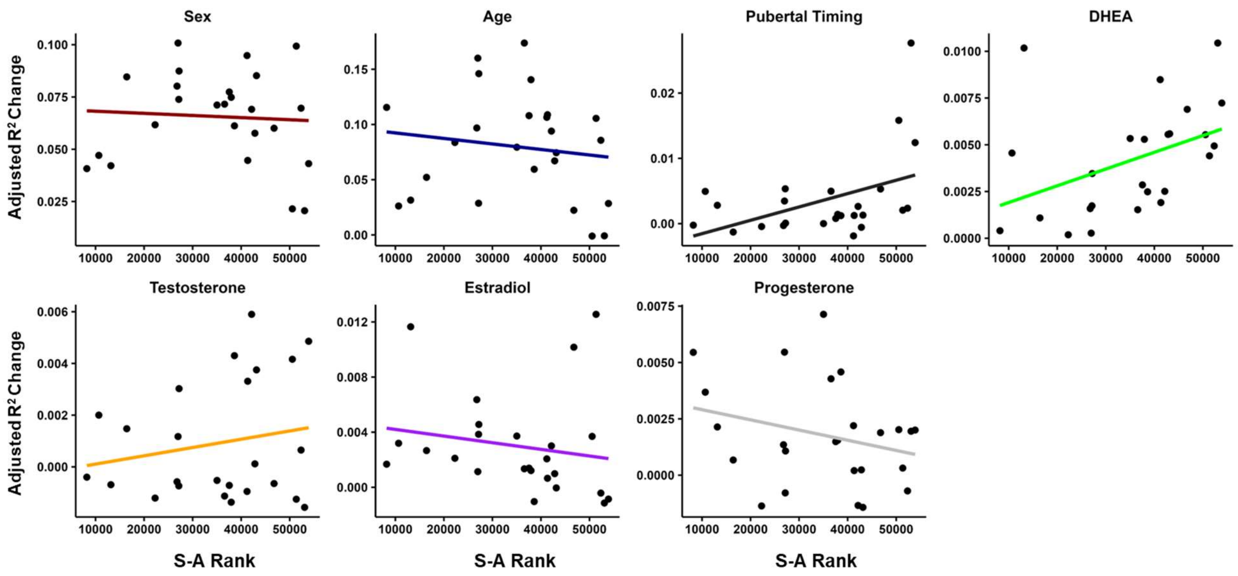

Supplemental Figure 3. Relationships between the FA variance explained by pubertal terms and the sensorimotor-association axis. The fitted regression lines with the individual datapoints for the relationships between the adjusted R^2 change for each model and the S-A value for each term are shown. The fitted lines are the same as shown in Figure 5, however, these plots contain the individual datapoints as well to better visualize these relationships.

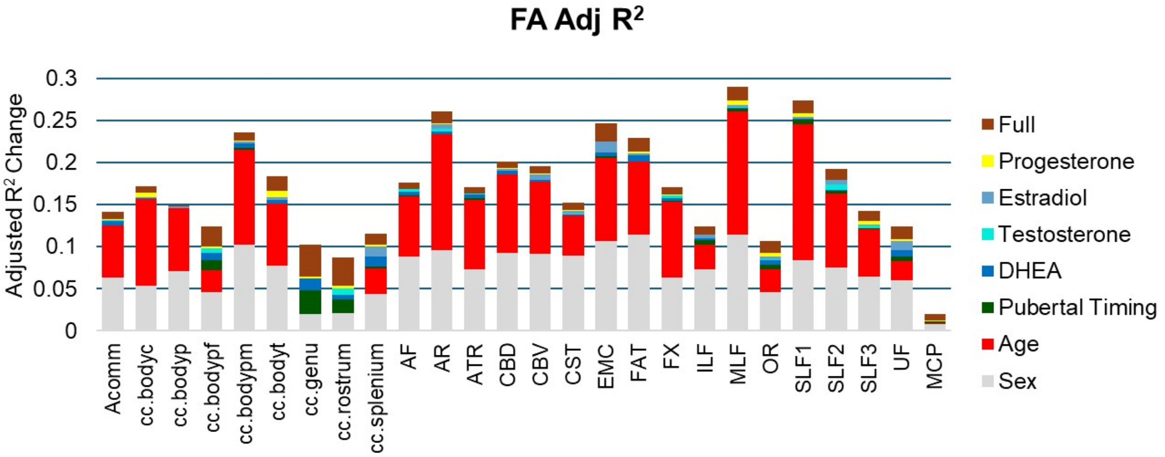

Supplemental Figure 4. Age-adjusted Hormone Models: FA adjusted R^2 Model Differences. The proportion of variance explained by sex model was calculated with adjusted R^2. The change in adjusted R^2 was then calculated sequentially between the more complex model and the simpler model (e.g., Age-Sex, Puberty-Age, [DHEA, Testosterone, Estradiol, or Progesterone]-Puberty, and Full-Puberty). Sex and age accounted for the largest amount of additional variance. The addition of pubertal status and hormones accounted for a small amount of additional variance in the models for most tracts.

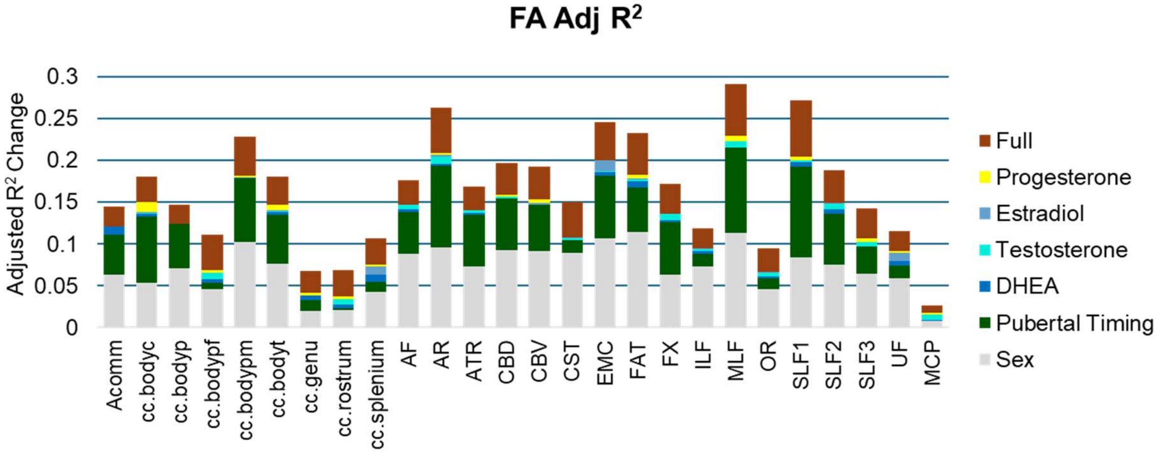

Supplemental Figure 5. Puberty-adjusted age hormone analysis: FA adjusted R^2 Model Differences. The proportion of variance explained by sex model was calculated with adjusted R^2. The adjusted R^2 change between the more complex model and the simpler model was calculated and is shown (e.g., Age-Sex, Pubertal Timing-Age, [DHEA, Testosterone, Estradiol, or Progesterone]-Pubertal Timing, and Full-Pubertal Timing). This analysis included pubertal variables in the model first, followed by including age in the full model. The additional R^2 analysis showed similar results as the main text (when age was added first). Progesterone added more variance in most networks than other hormones. The full model with age added a relatively large amount of variance to each region.

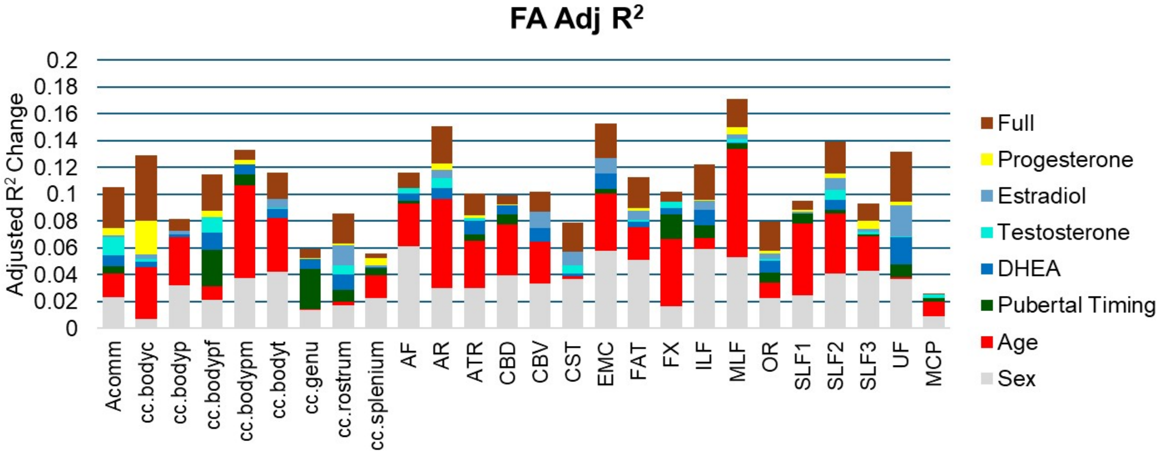

Supplemental Figure 6. Restricted age range analysis: FA adjusted R^2 Model Differences. The proportion of variance explained by sex model was calculated with adjusted R^2. The adjusted R^2 change between the more complex model and the simpler model was calculated and is shown (e.g., Age-Sex, Pubertal Timing-Age, [DHEA, Testosterone, Estradiol, or Progesterone]-Pubertal Timing, and Full-Pubertal Timing). We examined results from a sensitivity analysis in a restricted age range of 9-18 year olds. While less than the full sample, Age still explained a large amount unique variance in most tracts.

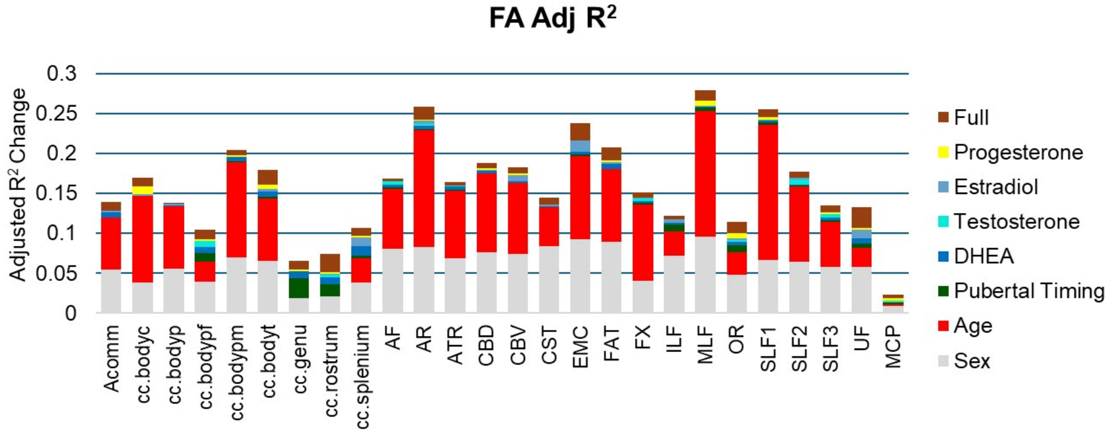

Supplemental Figure 7. No birth control subset: FA adjusted R^2 Model Differences. Excluding individuals on birth control changed the best fitting model for some tracts. The additional variance explained by each model remained similar to the results from the main text.

SI References

Dale, A. M., Fischl, B., & Sereno, M. I. (1999). Cortical surface-based analysis. I. Segmentation and surface reconstruction. *Neuroimage*, *9*(2), 179-194. https://doi.org/10.1006/nimg.1998.0395

Elam, J. S., Glasser, M. F., Harms, M. P., Sotiropoulos, S. N., Andersson, J. L. R., Burgess, G. C.,…Van Essen, D. C. (2021). The Human Connectome Project: A retrospective. *Neuroimage*, *244*, 118543. https://doi.org/10.1016/j.neuroimage.2021.118543

Fischl, B., & Dale, A. M. (2000). Measuring the thickness of the human cerebral cortex from magnetic resonance images. *Proc Natl Acad Sci U S A*, *97*(20), 11050-11055. https://doi.org/10.1073/pnas.200033797

Glasser, M. F., Coalson, T. S., Robinson, E. C., Hacker, C. D., Harwell, J., Yacoub, E.,…Van Essen, D. C. (2016). A multi-modal parcellation of human cerebral cortex. *Nature*, *536*(7615), 171-178. https://doi.org/10.1038/nature18933

Glasser, M. F., Sotiropoulos, S. N., Wilson, J. A., Coalson, T. S., Fischl, B., Andersson, J. L.,…Consortium, W. U. M. H. C. P. (2013). The minimal preprocessing pipelines for the Human Connectome Project. *Neuroimage*, *80*, 105-124. https://doi.org/10.1016/j.neuroimage.2013.04.127

Glasser, M. F., & Van Essen, D. C. (2011). Mapping human cortical areas in vivo based on myelin content as revealed by T1- and T2-weighted MRI. *J Neurosci*, *31*(32), 11597-11616. https://doi.org/10.1523/JNEUROSCI.2180-11.2011

Harms, M. P., Somerville, L. H., Ances, B. M., Andersson, J., Barch, D. M., Bastiani, M.,…Yacoub, E. (2018). Extending the Human Connectome Project across ages: Imaging protocols for the Lifespan Development and Aging projects. *Neuroimage*, *183*, 972-984. https://doi.org/10.1016/j.neuroimage.2018.09.060

Herting, M. M., Uban, K. A., Gonzalez, M. R., Baker, F. C., Kan, E. C., Thompson, W. K.,…Sowell, E. R. (2020). Correspondence Between Perceived Pubertal Development and Hormone Levels in 9-10 Year-Olds From the Adolescent Brain Cognitive Development Study. *Front Endocrinol (Lausanne)*, *11*, 549928. https://doi.org/10.3389/fendo.2020.549928

Morris, N. M., & Udry, J. R. (1980). Validation of a self-administered instrument to assess stage of adolescent development. *J Youth Adolesc*, *9*(3), 271-280. https://doi.org/10.1007/BF02088471

Omary, A., Curtis, M., Cheng, T. W., Mair, P., Shirtcliff, E. A., Barch, D. M., & Somerville, L. H. (2025). Multimodal Measurement of Pubertal Development: Stage, Timing, Tempo, and Hormones. *Child Dev*. https://doi.org/10.1111/cdev.14220

Petersen, A. C., Crockett, L., Richards, M., & Boxer, A. (1988). A self-report measure of pubertal status: Reliability, validity, and initial norms. *J Youth Adolesc*, *17*(2), 117-133. https://doi.org/10.1007/BF01537962

Robinson, E. C., Jbabdi, S., Glasser, M. F., Andersson, J., Burgess, G. C., Harms, M. P.,…Jenkinson, M. (2014). MSM: a new flexible framework for Multimodal Surface Matching. *Neuroimage*, *100*, 414-426. https://doi.org/10.1016/j.neuroimage.2014.05.069

Shirtcliff, E. A., Dahl, R. E., & Pollak, S. D. (2009). Pubertal development: correspondence between hormonal and physical development. *Child Dev*, *80*(2), 327-337. https://doi.org/10.1111/j.1467-8624.2009.01263.x

Tisdall, M. D., Reuter, M., Qureshi, A., Buckner, R. L., Fischl, B., & van der Kouwe, A. J. W. (2016). Prospective motion correction with volumetric navigators (vNavs)

reduces the bias and variance in brain morphometry induced by subject motion. *Neuroimage*, *127*, 11-22. https://doi.org/10.1016/j.neuroimage.2015.11.054
